# The olfactory epithelium as a port of entry in neonatal neurolisteriosis

**DOI:** 10.1038/s41467-018-06668-2

**Published:** 2018-10-15

**Authors:** Dennis Pägelow, Chintan Chhatbar, Andreas Beineke, Xiaokun Liu, Andreas Nerlich, Kira van Vorst, Manfred Rohde, Ulrich Kalinke, Reinhold Förster, Stephan Halle, Peter Valentin-Weigand, Mathias W. Hornef, Marcus Fulde

**Affiliations:** 1 Institute for Microbiology, University of Veterinary Medicine Hannover, D-30173 Hannover, Germany; 2 Institute of Microbiology and Epizootics, Centre for Infection Medicine, Freie Universität Berlin, D-14163 Berlin, Germany; 3 Institute for Experimental Infection Research, TWINCORE, Centre for Experimental and Clinical Infection Research, a joint venture between the Hannover Medical School and the Helmholtz Centre for Infection Research, D-30625 Hannover, Germany; 4 Institute for Pathology, University of Veterinary Medicine, D-30559 Hannover, Germany; 5 Institute of Immunology, Hannover Medical School, D-30625 Hannover, Germany; 6 Department of Internal Medicine/Infectious Diseases and Pulmonary Medicine, Charité Universitätsmedizin Berlin, D-10117 Berlin, Germany; 7 Central Facility for Microscopy, Helmholtz Centre for Infection Research, D-38124 Braunschweig, Germany; 8 Institute of Medical Microbiology, University Hospital RWTH Aachen, D-52074 Aachen, Germany

## Abstract

Bacterial infections of the central nervous system (CNS) remain a major cause of mortality in the neonatal population. Commonly used parenteral infection models, however, do not reflect the early course of the disease leaving this critical step of the pathogenesis largely unexplored. Here, we analyzed nasal exposure of 1-day-old newborn mice to *Listeria monocytogenes* (*Lm*). We found that nasal, but not intragastric administration, led to early CNS infection in neonate mice. In particular, upon bacterial invasion of the olfactory epithelium, *Lm* subsequently spread along the sensory neurons entering the brain tissue at the cribriform plate and causing a significant influx of monocytes and neutrophils. CNS infection required listeriolysin for penetration of the olfactory epithelium and ActA, a mediator of intracellular mobility, for translocation into the brain tissue. Taken together, we propose an alternative port of entry and route of infection for neonatal neurolisteriosis and present a novel infection model to mimic the clinical features of late-onset disease in human neonates.

## Abstract (web summary)

*Listeria monocytogenes* causes meningitis in newborns. Here, Pägelow et al. present a mouse model of neonatal cerebral listeriosis, and show that nasal inoculation, but not intragastric administration, leads to early brain infection in the absence of bacteraemia during the neonatal period.

## Introduction

Bacterial infections of the central nervous system (CNS) represent an important cause of morbidity and mortality worldwide. Particularly newborns are affected with significant long-term neurological sequelae such as cognitive impairment and developmental retardation as well as high mortality rates. Neonatal bacterial meningitis is predominantly attributed to group B streptococci (*Streptococcus agalactiae*), *Escherichia coli* K1 and *Listeria monocytogenes* (*Lm*), together accounting for ~70–80% of cases in industrialized countries^1,2^. Although preventive measures such as improved hygiene regimens and antibiotic intrapartum prophylaxis have reduced the incidences in the last decades, the mortality associated with neonatal meningitis still remains substantial^3^.

Infections of newborns with *Lm*, referred to as neonatal listeriosis, are subdivided in an early-onset disease (EOD) and a late-onset (LOD) form. EOD manifests within the first days of life and is most likely acquired prenatally by aspiration of contaminated amniotic fluid^4–6^. In contrast, LOD develops several days after birth and is thought to be transmitted during parturition through contact with the maternal vaginal or intestinal microbiota. It usually manifests as meningitis or meningoencephalitis with high cell counts in the cerebrospinal fluid^3,7,8^. Septicemia is less common in LOD and the route of the infectious agent from the port of entry to the central nervous system is largely unknown.

*Lm* represents an important food-borne pathogen and is found ubiquitously in the environment. In vitro and in vivo studies have shown that *Lm* is able to (i) overcome the intestinal epithelial barrier, (ii) survive and replicate within the subepithelial tissue and systemic circulation and, finally, (iii) reach the CNS. *Lm* is generally thought to reach the CNS by penetration of the blood-brain and blood-CSF barriers either by receptor-mediated invasion, or as a cargo of migratory pro-inflammatory immune cells^9–12^. In contrast, *Lm* in ruminants was suggested to reach the CNS also non-hematogenously, ascending via cranial nerves^13,14^.

Here we systematically analyzed the potential role of the nasopharyngeal mucosa as entry port for CNS infection including a characterization of the local host response and involved bacterial virulence factors. Employing a novel nasal *Lm* infection model in neonate mice, we demonstrate (i) colonization of the neonatal nasopharyngeal mucosa independent of the bacterial adhesions InlA and InlB, (ii) bacterial invasion of the olfactory epithelium and disruption of epithelial integrity dependent on the bacterial virulence factor listeriolysin (LLO), (iii) bacterial association with nerve cell structures, ascending infection and penetration of the cribriform plate in a *Lm* ActA dependent manner, and finally, (iv) initial infection of the frontal brain segments with significant cytokine induction and immune cell recruitment and subsequent bacterial spread to other brain segments in the course of the infection. Our results illustrate the particular susceptibility of the neonate host to CNS infection. The occurrence of CNS infections in the absence of bacteremia in the majority of LOD cases suggests that a similar route of infection may also apply to the infection in human neonates.

## Results

### Nasal exposure leads to early cerebral spread

**Fig. 1:**
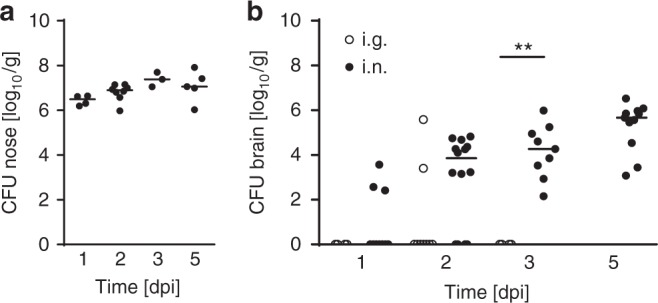
Spread of *Lm* to CNS tissue after nasal administration. Bacterial counts in nasopharyngeal mucosal tissue (**a**) and total brain tissue (**b**) at 1, 2, 3 and 5 days post infection (dpi). One-day-old mice were infected with either 1 × 10^7^ CFU intragastrically (○, i.g.) or 1 × 10^4^ CFU intranasally (●, i.n.). Each dot defines an individual mouse. Nose: *n* = 4 (1 dpi), *n* = 8 (2 dpi), *n* = 3 (3 dpi), *n* = 5 (5 dpi); brain i.n.: *n* = 10 (1 dpi), *n* = 14 (2 dpi), *n* = 9 (3 dpi), *n* = 10 (5 dpi); brain i.g.: *n* = 4 (1 dpi), *n* = 9 (2 dpi), *n* = 4 (3 dpi); median; unpaired, two-tailed Mann–Whitney test; **p < 0.01

We here evaluated the situation of the human neonate during parturition exposed to the maternal vaginal and enteric microbiota and at risk to acquire *Lm*-mediated LOD. We exposed the nostrils of 1-day-old mice with 1 × 10^4^ CFU *Lm*. As a control, 1 × 10^7^ CFU *Lm* was administered by oral gavage. Oral gavage prevented exposure of the upper respiratory airways and ensured administration of the bacteria into the gastrointestinal tract. Nasal administration led to a stable colonization of the newborn's nasal cavity over at least 5 days (Fig. 1a), consistent with the observation by Becroft et al. who reported the isolation of *Lm* from nasal swabs of human neonates suffering from listeriosis^15^. Unexpectedly, the bacterial count in cerebral tissue at day 1, 2, 3 and 5 dpi indicated a rapid spread of *Lm* to the CNS following nasal exposure. Ten out of 14 animals (72%) exhibited a positive culture in total brain tissue already at 2 dpi and 100% of the mice at 3 and 5 dpi. In contrast, only 2 out of 15 animals (15%) exhibited a positive culture in total brain tissue after intragastric administration of *Lm* (Fig. 1b). These results suggest the existence of a direct respiratory route of infection that is independent of bacteremia. Indeed, bacterial culture from blood samples obtained at day 1, 2, 3 and 5 after nasal administration revealed a positive result only in a minority of animals (4 out of 11, 36%) and only at a late time point after infection (Supplementary Figure 1). Most notably, the direct respiratory route of infection with early CNS involvement showed a marked age-dependency. Nasal exposure of 11-day-old mice still resulted in nasal colonization, but failed to induce CNS infection at 1 or 3 dpi (Supplementary Figure 2A and B).

**Fig. 2:**
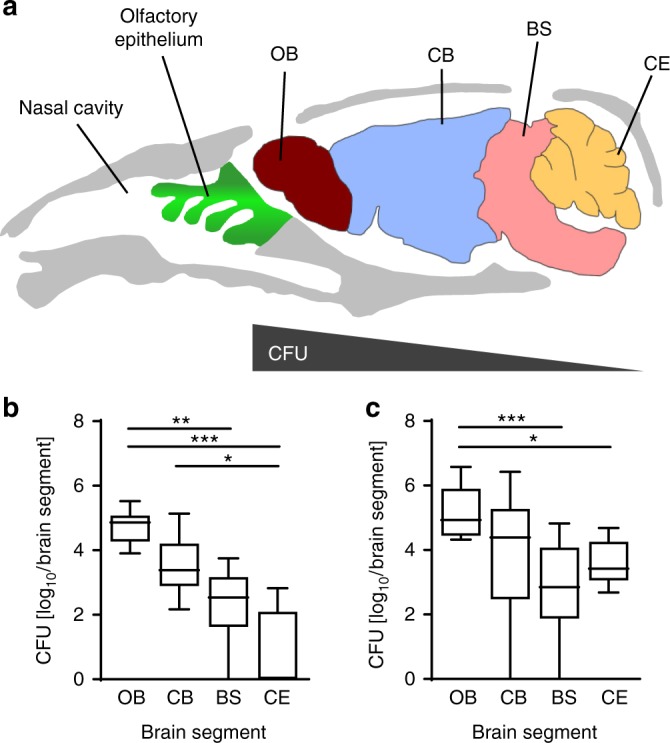
Cerebral infection spreads along the rostro-caudal axis. **a** Sagittal section of a mouse skull with nasal cavity, olfactory epithelium and the different brain segments such as the olfactory bulb (OB), cerebrum (CB), brain stem (BS), and cerebellum (CE). **b** and **c** Bacterial counts in the respective brain segments of 1-day-old neonatal mice infected i.n. with 1 × 10^4^ CFU *Lm* at 3 (**b**) and 5 (**c**) dpi. Total brain tissue was collected after transcardial perfusion and dissected into OB, CB, BS, and CE as indicated. The Data set at 3 dpi is derived from *n* = 8 and at 5 dpi derived from *n* = 10 pups. Box and whisker; median and 5–95 percentile. One-way ANOVA Kruskal-Wallis with Dunnets’ post test; *p < 0.05; **p < 0.01; ***p < 0.001

In order to test the hypothesis of a non-hematogenous direct route of infection through the respiratory subepithelial tissue in newborn animals, we next determined the bacterial burden in the different brain segments, the olfactory bulb (OB), the cerebrum (CB), the brain stem (BS), and the cerebellum (CE) at 3 and 5 days after nasal infection. The brains were removed following transcardial perfusion with PBS to avoid false positive results from intravascular bacteria. Subsequently, the different brain segments were dissected and analyzed by bacterial culture. As depicted in Fig. 2, significantly more bacteria were isolated from the frontal OB than from the caudal BS and CE at 3 dpi (Fig. 2b). A similar observation was made after 5 dpi (Fig. 2c), but the frontal-occipital CFU gradient was less pronounced, suggesting intracranial dissemination of *Lm* from the frontal segment to other parts of the brain parenchyma during the course of the infection.

### *Lm* invades the olfactory epithelium

**Fig. 3:**
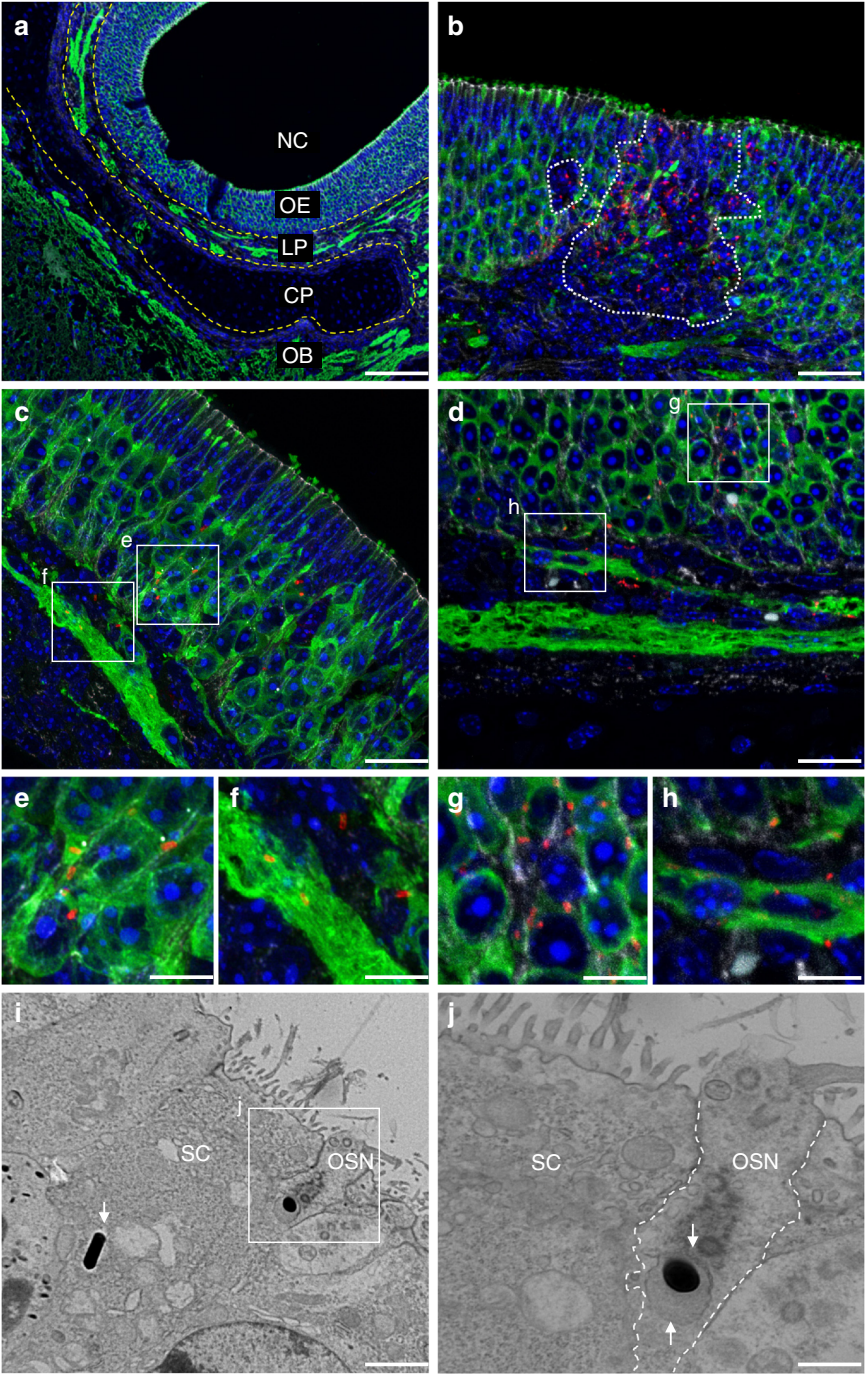
*Lm* invades the olfactory mucosa. **a**–**h** Immunohistochemistry (IHC) and **i** and **j** transmission electron microscopy (TEM) of the olfactory mucosa of 1-day-old mice infected with 1 × 10^7^ CFU *Lm* i.n. or age-matched control animals. For IHC, sections were stained for *Lm* (red), the neuronal marker β-tubulin III (green), the tight-junction marker β-catenin (white), and DNA (DAPI, blue). **a** Sagittal view of the olfactory system of a 2-day-old control mouse. Nasal cavity (NC), olfactory epithelium (OE), *lamina propria* (LP), olfactory bulb (OB), and cribriform plate (CP). **b**–**h** IHC of the OE and subepithelial mucosal tissue after i.n. at 1 dpi. Zoomed views of intraepithelia **e** and **g**  as well as **f** and **h** nerve-associated bacteria in the LP. Disruption of the OE is marked with dotted lines **b** (**a**: scale bar 100 µm, **b**–**d**: scale bar 20 µm, **e**–**h**: scale bar 5 µm). **i** and **j** TEM of the OE at 12 hpi, showing *Lm* within a sustentacular cell (SC; white arrow) and an olfactory sensory neuron (OSN; framed insert, white arrow). *Lm* is surrounded by a vacuolar structure within the OSN (white arrows in **j**) (**i**: scale bar 2 µm, **j**: scale bar 1 µm). IHC and TEM images are representative for samples from each *n* = 8 pups; images of age-matched controls are derived from *n* = 8 pups

Next, we characterized the route of transmigration from the nasal cavity (NC) to the frontal brain tissue. Figure 3a provides a histological overview of the anatomical structures in the distal NC and OB. The NC is lined by the highly ciliated respiratory epithelium (not stained) or the β-tubulin III positive olfactory epithelium (OE). The OE covers the *lamina propria* (LP), in which axons of the β-tubulin III positive olfactory sensory neurons are bundled to nerves. The nerves project through the cartilaginous cribriform plate (CP) reaching the frontal CNS, where they form the nerve fiber layer of the OB. To characterize the translocation of *Lm* through the olfactory epithelium and the subsequent spread into the brain, we infected 1-day-old newborn mice and obtained tissue for immunostaining and transmission electron microscopy. Twelve hours or 1 dpi, mice were killed and sagittally dissected skull tissue was prepared. As illustrated in Fig. 3b–h, bacteria were detected inside olfactory epithelial cells and the subepithelial tissue. Two different phenotypes were observed. First, single bacteria were found in close contact to β-tubulin III positive neuronal structures within the epithelium (Fig. 3c–e, g). Z-stack images indicated that the majority of these bacteria were localized intracellularly in neurons (Supplementary Figure 3). Single bacteria were also found below the olfactory epithelium within the *lamina propria*, again in close contact with neuronal axon bundles (Fig. 3f, h). Second, large groups of bacteria were observed within the olfactory epithelium and *lamina propria* accompanied by significant tissue damage, as indicated by a loss of the immunofluorescence signal (dotted line in Fig. 3b, Supplementary Figure 4A–C). For these bacterial groups, no association with neuronal cell structures was observed.

The olfactory epithelium mainly consists of two cell types: olfactory sensory neurons (OSNs), which protrude with their hair-like structures into the NC and supporting sustentacular cells (SC) (Fig. 3i, j). *Lm* was readily detected in both cell types (Fig. 3i, j). In accordance with the described intracellular lifestyle of *Lm*, bacteria were similarly detected in an intracellular compartment, surrounded by a membranous structure (white arrows in Fig. 3j), but also free in the cell cytosol (white arrow in Fig. 3i). However, the intracellular localization was not systematically examined.

### *Lm* associates with olfactory sensory nerve fibers

**Fig. 4:**
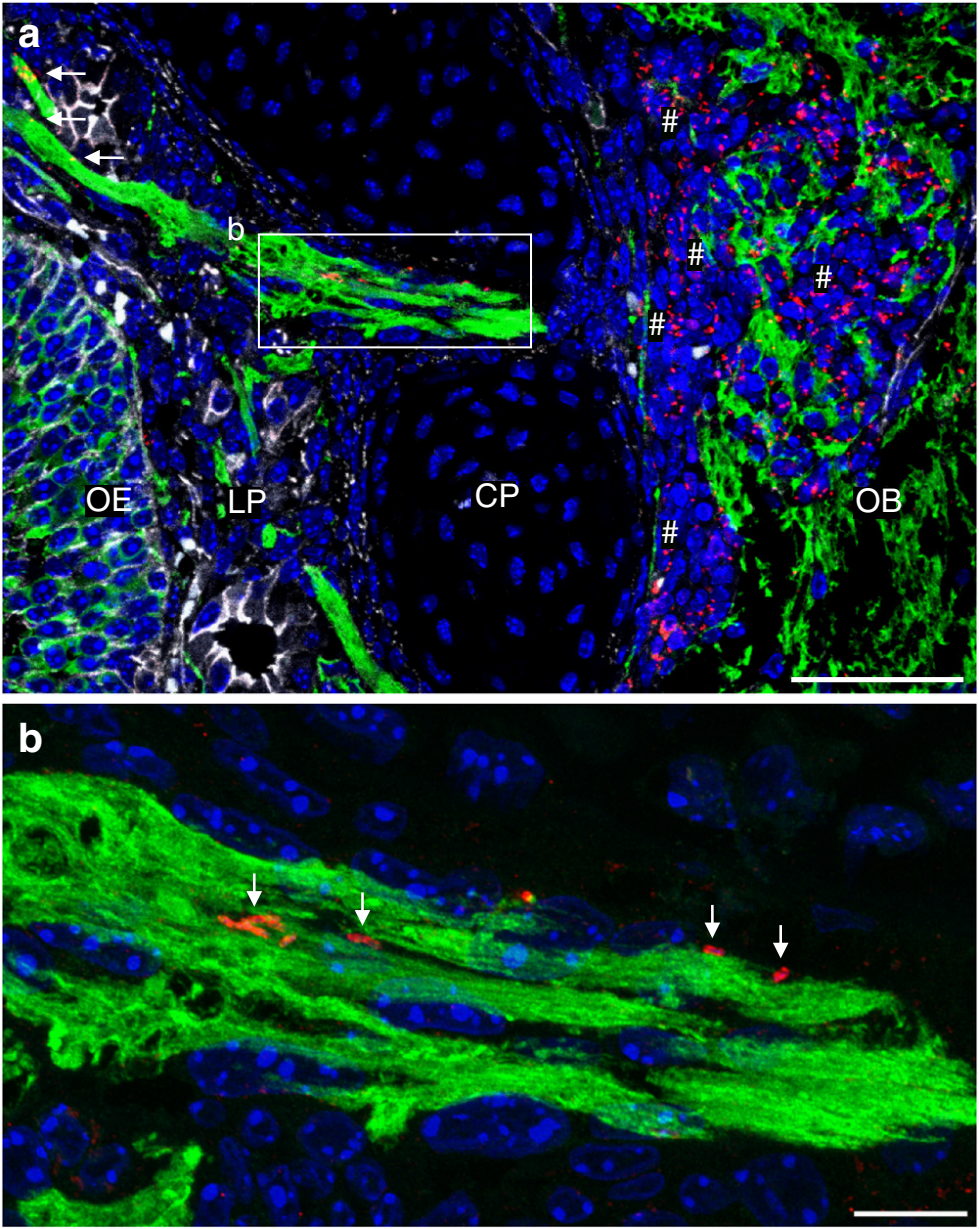
*Lm* is situated in close proximity to olfactory nerves. Immunostaining of the olfactory tissue after i.n. infection of 1-day-old mice with 1 × 10^7^ CFU *Lm* at 1 dpi. Olfactory bulb (OB), cribriform plate (CP), *lamina propria* (LP), and olfactory epithelium (OE). For IHC, sections were stained for *Lm* (red), the neuronal marker β-tubulin III (green), the tight-junction marker β-catenin (white) and DNA (DAPI, blue). **a** Sagittal view of the olfactory tissue. Bacteria are found associated with nerves in the LP (white arrows) and the cribriform plate (framed insert) as well as within the CNS (white hash symbols) (scale bar 50 µm). **b** Enlarged insert from **a** showing *Lm* (red, white arrows) in close proximity to axons (green) in the cribriform plate (scale bar 10 µm). The images are representative of *n* = 8 pups

**Fig. 5:**
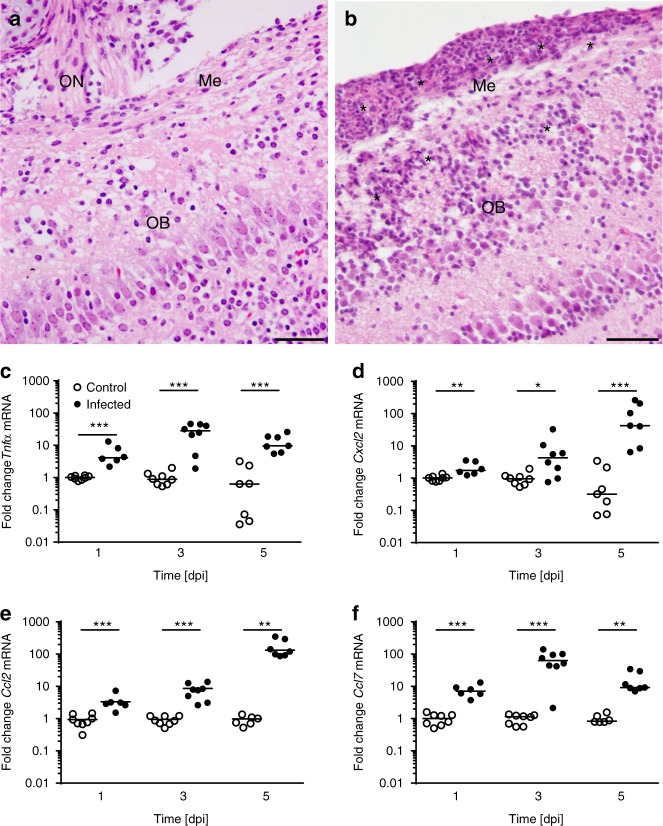
Intranasal infection with *Lm* induces inflammation in the CNS. One-day-old mice were infected i.n. with 1 × 10^4^ CFU *Lm* or left untreated. Tissue was collected at 1, 3, and 5 dpi or from age-matched control animals. **a** Sagittal view of the olfactory system of a 6-day-old control mouse. HE-staining of olfactory nerve (ON), meninges (Me), olfactory bulb (OB). **b** Sagittal view of the OB at 5 dpi, asterisks mark inflammatory infiltrates. Images are representative for samples from *n* = 6 pups; images of age-matched controls are derived from *n* = 4 pups (scale bar 50 µm). **c**–**f** For analyses of cytokine expressions, whole-brain tissues were collected at the indicated time points after infection or from age-matched control animals and total RNA was prepared. qRT–PCR for **c** *Tnfα*, **d** *Cxcl2*, **e** *Ccl2*, and **d** *Ccl7*. Transcript results were normalized to the housekeeping gene *Hprt* and expressed as fold change. Each dot defines an individual mouse. Control animals: *n* = 8 (1 dpi), *n* = 8 (3 dpi), *n* = 7 (5 dpi); infected animals: *n* = 6 (1 dpi), *n* = 8 (3 dpi), *n* = 7 (5 dpi); median; unpaired, two-tailed Mann–Whitney test; *p < 0.05, **p < 0.01, ***p < 0.001

We next focused on the further bacterial spread from the tissue underlying the olfactory epithelium. Immunostaining visualized bacteria in close proximity to nerve fibers projecting through the cribriform plate (Fig. 4a, b). Subsequently, *Lm* proliferated within the adjacent intracranial tissue and started to disseminate into the brain parenchyma (white hash symbols in Fig. 4a). Infection of the CNS tissue resulted in local histopathological tissue alterations and strong induction of pro-inflammatory cytokines and chemokines. Histopathological analysis revealed a strong influx of immune cells into the meninges and the brain parenchyma, characterizing a purulent-necrotizing meningitis and meningoencephalitis (Fig. 5a, b). Consistently, quantitative RT–PCR revealed a significant increase in *Tnfa* (Fig. 5c) and *Cxcl2* (Fig. 5d) mRNA in total brain tissue of intranasally infected neonate mice at 1, 3, and 5 dpi as compared to uninfected age-matched controls. The early increase in cytokine expression correlated with the detection of *Lm* within intracranial tissue (Fig. 4a). Also, mRNA expression of the monocyte-attracting chemokines *Ccl2* (Fig. 5e) and *Ccl7* (Fig. 5f) was significantly increased consistent with their documented role in the innate immune responses to *Lm*^16^.

### Characterization of recruited immune cells

**Fig. 6:**
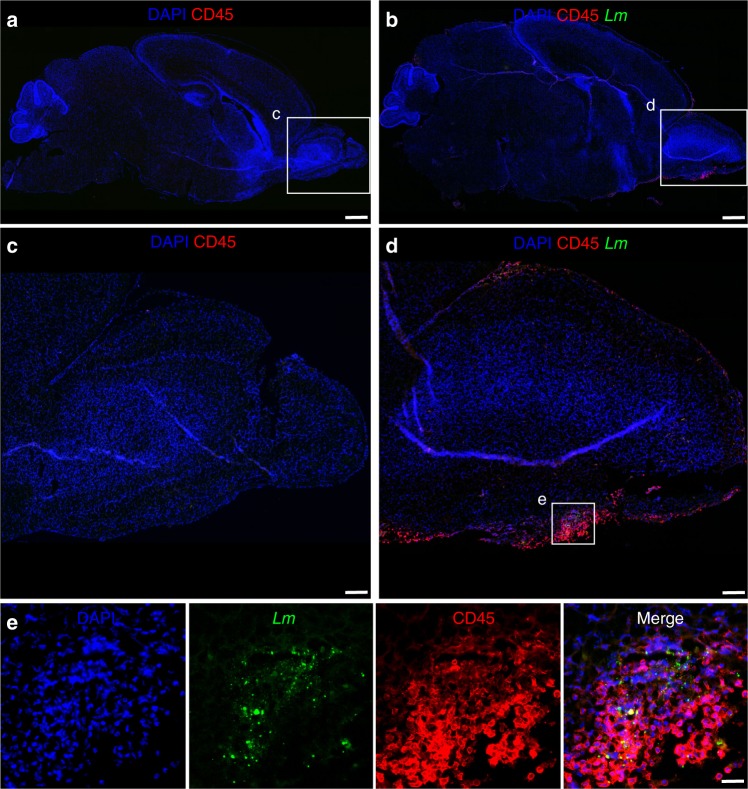
Accumulation of immune cells in the CNS. One-day-old mice were either infected i.n. with 1 × 10^5^ CFU *Lm* **b**, **d**, **e**  or exposed to sterile PBS **a**, **c**. Tissue sections were stained for *Lm* (green), the leukocyte marker CD45 (red), and DNA (DAPI, blue). Sagittal overview of entire brain tissue of an age-matched control mouse (**a**) and after i.n. infection at 3 dpi (**b**) (scale bar 500 µm). Respective zoomed view of the olfactory bulb (**c**) and (**d**) (scale bar 100 µm). **e** Zoomed view from **d** (scale bar 20 µm). DAPI (blue), *Lm* (green), and CD45 (red) are shown separately (first three panels) and as a merge image. Images are representative for samples from *n* = 6 pups; images of age-matched controls are derived from *n* = 6 pups

To further characterize the *Lm*-induced cerebral immune response, brain tissue sections of infected and age-matched control mice were stained using the leukocyte marker CD45 (red), and counterstained against *Lm* (green) and DNA (blue). As expected, CD45^+^ cells accumulated at the interphase of the OB with the basal meninges upon *Lm* infection extending into the intact brain parenchyma in the frontal part of the OB. In contrast, CD45^+^ cells were largely absent from uninfected control tissue (Fig. 6a–d). Higher magnification of the basal OB illustrated the close anatomical association of CD45^+^ and CD45^+^CD11b^+^ positive cells with tissue-infiltrating bacteria (Fig. 6e and Supplementary Figure 5).

**Fig. 7:**
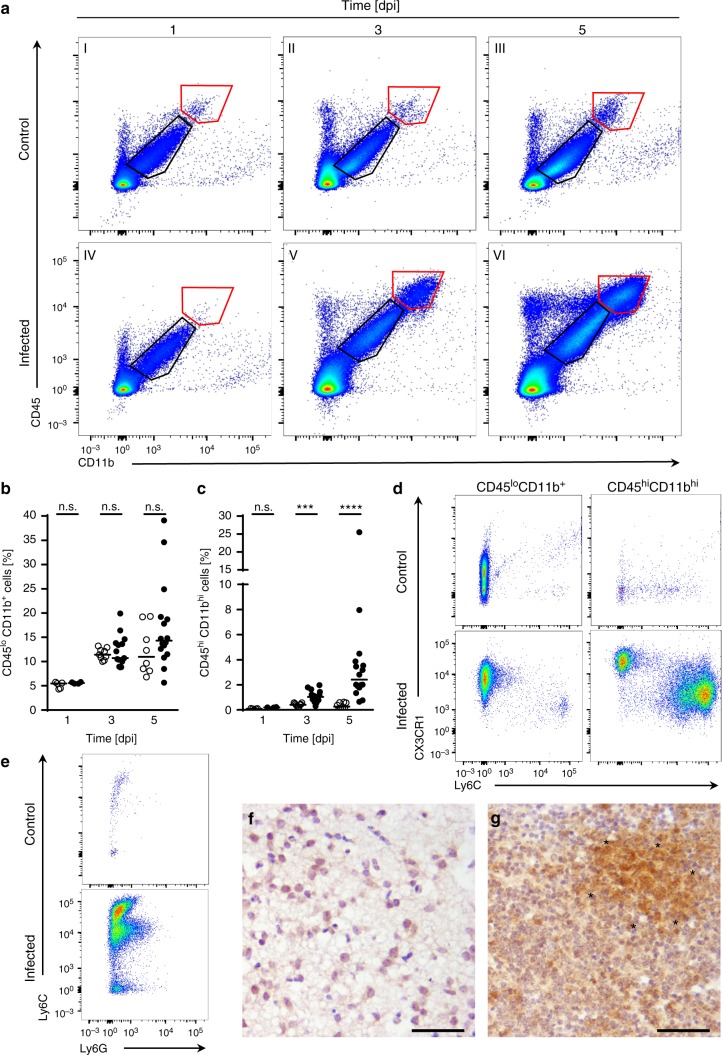
Influx of CD45^+^CD11b^+^ cells during neurolisteriois. One-day-old mice were either infected i.n. with 1 × 10^4^ *Lm* (●) or exposed to sterile PBS (**○**) **a**–**g**. For flow cytometry, total brain tissues were collected at 1, 3 and 5 dpi after transcardial perfusion with PBS and processed accordingly. **a** Representative FACS dot plots of age-matched control (I–III) and *Lm* infected (IV–VI) animals at 1, 3 and 5 dpi as indicated. Cells were gated on the common leukocyte marker CD45 and the pan-myeloid marker CD11b. Percent of **b** CD45^lo^CD11b^+^ and **c** CD45^hi^CD11b^hi^ cells. Each dot defines an individual mouse. Control animals: *n* = 5 (1 dpi), *n* = 9 (3 dpi), *n* = 8 (5 dpi); infected animals: *n* = 4 (1 dpi), *n* = 15 (3 dpi), *n* = 16 (5 dpi). Median; unpaired, two-tailed Mann–Whitney test; n.s. *p* > 0.05, **p < 0.01, ***p < 0.001; ****p < 0.0001. **d** Representative dot plots of the cell populations indicated in **b** and **c**, additionally examined for their expression of CX3CR1 and Ly6C at 5 dpi. **e** Representative dot plot of CD45^hi^CD11b^hi^ cells gated on Ly6C and the neutrophil marker Ly6G. For IHC, whole brains of age-matched control (**f**) or i.n.-infected mice (**g**) were stained for Ly6G at 5 dpi. Images are representative for samples from *n* = 6 pups; images of age-matched controls are derived from *n* = 4 pups (scale bar 50 µm)

Using flow cytometric analysis, we next characterized the infiltrating cells in more detail. To avoid contamination with intravascular immune cells, mice were transcardially perfused with sterile PBS prior to tissue sampling and enzymatic processing. Two major cell subsets based on expression of the common leukocyte marker CD45 and the pan-myeloid marker CD11b were analyzed. As depicted in Fig. 7a (black pentagon), CD45^lo^CD11b^+^ cells, which mainly consist of microglia^17–20^, represent the largest of the cell populations analyzed. The number of CD45^lo^CD11b^+^ cells increased with age but remained largely independent of infection (Fig. 7b). Neutrophils, monocytes and monocyte-derived macrophages can be summarized under a CD45^hi^CD11b^hi^ expression pattern^17,19,21–23^. In contrast to the CD45^lo^CD11b^+^ population, CD45^hi^CD11b^hi^ cells were significantly increased upon *Lm* infection at 3 dpi with 1.03% vs. 0.41% in control animals (Fig. 7c). At 5 dpi, the CD45^hi^CD11b^hi^ population reached 2.42% of the total cell count (range 0.65–25.5%) accounting for an almost tenfold increase compared to control animals (0.26%; range 0.076–0.62%). Notably, the CD45^hi^CD11b^hi^ cell population did not increase with age as seen for CD45^lo^CD11b^+^ cells. To further dissect both cell populations, we additionally stained for the surface markers CX3CR1 and Ly6C. CD45^lo^CD11b^+^ consistently stained positive for CX3CR1, but remained negative for Ly6C. In contrast, the CD45^hi^CD11b^hi^ population fell into two distinct populations, namely a CX3CR1^+^Ly6C^−^ and a CX3CR1^−^Ly6C^+^ population at 5 dpi (Fig. 7d). Both populations were only observed in infected tissue. We also included the neutrophil marker Ly6G in our analysis and gated the CD45^hi^CD11b^hi^ cell population for both, Ly6G and Ly6C. As depicted in Fig. 7e, three populations of Ly6C cells (high, int and low) were detected in brain homogenates of infected mice. Notably, a significant positive Ly6G signal was only detected for Ly6C^int^ cells, suggesting a role of neutrophils in the course of neonatal neurolisteriosis. Consistently, a significant cellular influx of Ly6G positive cells into the brain parenchyma of infected, but not uninfected mice, was also observed by immunohistochemistry (Fig. 7f, g).

### Contribution of bacterial virulence factors

**Fig. 8:**
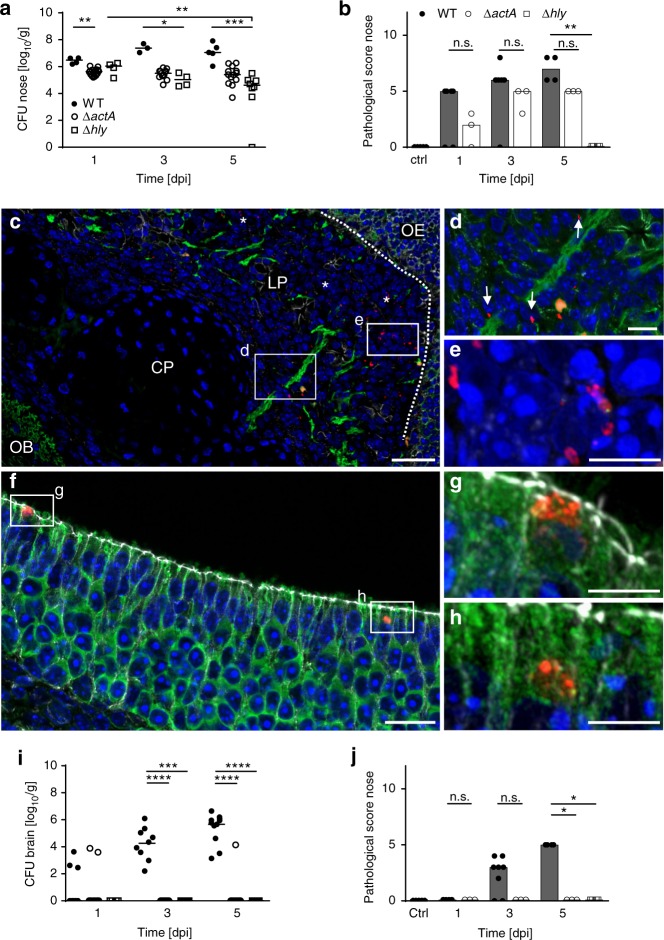
LLO and ActA are essential for CNS invasion. Bacterial counts in **a** nose and **i** brain after i.n. infection of 1-day-old mice with 1 × 10^4^ CFU wild-type *Lm* (WT) an isogenic ActA-deficient (Δ*actA*) *Lm* strain and an isogenic LLO deficient (Δ*hly*) *Lm* strain at 1, 3, and 5 dpi. WT *Lm*: *n* = 4 (1 dpi), *n* = 3 (3 dpi), *n* = 5 (5 dpi); Δ*actA Lm*: *n* = 14 (1 dpi), *n* = 13 (3 dpi), *n* = 13 (5 dpi), Δ*hly Lm*: *n* = 4 (1 dpi), *n* = 4 (3 dpi), *n* = 8 (5 dpi). Median; ANOVA Kruskal-Wallis with Dunnets’ post test and for comparison of Δ*hly* *Lm* 1 dpi vs. Δ*hly Lm* 5 dpi unpaired, two-tailed Mann–Whitney test; n.s. *p* > 0.05; *p < 0.05; **p < 0.01; ***p < 0.001; ****p < 0.0001. Histopathological score from HE stained nasopharyngeal tissue (**b**) and total brain tissue (**j**) as specified in Methods section. Each dot defines an individual mouse. Control animals: *n* = 5; WT *Lm*: *n* = 6 (1 dpi), *n* = 8 (3 dpi), *n* = 4 (5 dpi); Δ*actA Lm*: *n* = 3 (1 dpi), *n* = 3 (3 dpi), *n* = 3 (5 dpi), Δ*hly Lm*: *n* = 4 (1 dpi). Median; unpaired, two-tailed Mann–Whitney test; n.s. *p* > 0.05; *p < 0.05; **p < 0.01. Immunostaining of the olfactory mucosa after i.n. infection of 1-day-old mice with 1 × 10^7^ CFU Δ*actA* (**c**–**e**) or Δ*hly* (**f**–**h**) *Lm*. Sections were stained for *Lm* (red), the neuronal marker β-tubulin III (green), the tight-junction marker β-catenin (white) and DNA (DAPI, blue). **c** Sagittal view of the olfactory mucosal tissue at 3 dpi with Δ*actA Lm* (scale bar 25 µm). White asterisks indicate subepithelial Δ*actA Lm*, dotted line illustrates disruption of the OE. **d** and **e** Zoomed view of the framed insert in **c**, white arrows indicate Δ*actA Lm* (**d**: scale bar 10 µm, **e**: scale bar 5 µm). **f** Sagittal view of the OE at 1 dpi with Δ*hly Lm* (scale bar 20 µm). **g** and **h** Zoomed view of the framed inserts in **f** (scale bar 5 µm). The images are representative for samples from each *n* = 6 pups

In addition to the internalins InlA and InlB, the actin assembly inducing protein ActA and the thiol-activated cholesterol-dependent cytolysin listeriolysin O (LLO, *hly*) represent established virulence factors of *Lm*^10,24–28^. To analyze their role in neonatal neurolisteriosis, wild-type *Lm* were analyzed in comparison to their isogenic *Lm* mutants. As shown in Supplementary Figure 6, an *Lm* InlAB double mutant colonized the nasal mucosa and translocated into the CNS tissue at levels comparable to wild-type bacteria (Fig. 1). This suggests that both InlA and InlB do not play a critical role during neonatal CNS infection. In contrast, infections with a Δ*actA* or Δ*hly Lm* mutant, led to significantly different bacterial organ counts and anatomical distribution as compared to wild-type infection. Colonization of the nasal mucosa was reduced for both Δ*actA* and Δ*hly* mutant *Lm* (Fig. 8a). Nevertheless, ActA-deficient bacteria still penetrated the olfactory epithelium causing mild focal to multifocal rhinitis (Fig. 8b and Supplementary Table 1). Notably, ActA-deficient *Lm* did not colocalize with neuronal cell structures as demonstrated for wild-type bacteria despite their presence in the LP (Fig. 8c, d). Also, ActA-deficient bacteria were mainly detected in bacterial aggregates (white asterisks in Fig. 8c; Supplementary Figure 7); whereas, the wild-type mainly appeared as single bacteria (Fig. 3b, d). Finally, invasion of the cerebral tissue was significantly reduced and histopathological changes of the brain tissue were completely absent following infection with *Lm* Δ*act*A bacteria (Fig. 8i, j; Supplementary Table 2). In contrast, LLO was required already during the early steps of the infection process. Although LLO deficient bacteria retained their ability to invade the olfactory epithelium at early time points, they failed to translocate and reach the LP leaving the cerebral tissue completely unaffected (Fig. 8f–j). These results indicate a critical contribution of ActA and LLO in the pathogenesis of neurolisteriosis with LLO being required for epithelial translocation and ActA for translocation into the cerebral tissue.

## Discussion

Bacterial CNS infections are associated with high mortality and significant long-term sequelae and remain a major health problem worldwide. This particularly accounts for neonates and young infants, the age group with the highest incidence of this type of infection. The reasons for the age-dependent susceptibility are not entirely clear but may reflect low colonization resistance, an impaired epithelial barrier or an immature immune system^29^. *Lm* belongs to the major causative agents of neonatal sepsis and meningitis and is characterized by a high mortality rate of up to 30%^3,6,30^. Although the risks associated with *Lm*-induced CNS infection have long been established, our knowledge about the underlying mechanisms is still incomplete. A major problem is the lack of a suitable neonatal infection model, which allows the analysis of the entire course of the infection, including bacterial colonization, mucosal tissue invasion, and organ spread as well as antibacterial immune activation.

Here, we analyzed nasal *Lm* infection of neonate mice. We demonstrate efficient colonization of the nasopharyngeal mucosa, invasion of the olfactory epithelium, bacterial spread within the subepithelial tissue, ascending infection in close proximity to nerve fibers, and penetration of the cribriform plate ultimately leading to infection of the frontal brain segments with significant cytokine induction and recruitment of inflammatory cells. Most notably, nasal exposure led to early CNS infection in all infected animals. Our results therefore suggest a novel route of *Lm*-induced CNS infection following respiratory exposure via translocation of the olfactory epithelium and penetration of the cribriform plate. These findings challenge the view that *Lm*-induced CNS infection in neonates occurs solely as a consequence of bacteremia following bacterial penetration of the intestinal mucosa^31^. Indeed, intragastric administration of *Lm* led to CNS infection, but only in a minority of newborn mice. This finding is consistent with previous reports on oral *Lm* infection in adult mice^32–34^. Even repeated administration over seven to ten consecutive days led to meningitis and rhombencephalitis in only 25–33 % of animals^35^. Thus, although bloodstream infection can lead to CNS infection, nasal exposure, spread through the olfactory epithelium and penetration through the cribriform plate appears to represent a particularly efficient route of *Lm*-mediated CNS infection.

Whereas human EOD is commonly thought to be acquired transplacentally or by aspiration of contaminated amniotic fluid^4,7,8^, LOD is transmitted during parturition from colonized maternal mucosal surfaces^7,8^. Prenatal disruption of the amniotic sac and the typical *occiput-anterior* position during passage of the birth canal expose the neonate to the maternal vaginal and enteric microbiota. Consistently, the causative agents of neonatal sepsis and meningitis, group B streptococci (GBS), *E. coli* K1 and *Lm*, are colonizers of the human´s vaginal or gastrointestinal tract. Also, *Lm* has been cultured from nasal swabs of infected newborns^15^. Our model mimics this mode of transmission by administering *Lm* nasally to 1-day-old mice. Strikingly, the resulting clinical picture resembles the human disease. Human LOD is associated with rapid progression and CNS infection, but a lower rate of septicemia^3^. Also in murine neonates, infection progressed rapidly with early involvement of the CNS but absence of significant bacteremia in the majority of animals. CNS infection was characterized by a strong pro-inflammatory reaction with influx of CD45^+^C11b^+^ immune cells to the site of infection. Similarly, a characteristic of human LOD is a high leukocyte count in the cerebrospinal fluid (CSF), which mainly consist of neutrophils and monocytes^3^. Consistently, we also observed a neutrophilic inflammation of the ventricles, which was accompanied by severe necrosis of the ependyma and an influx of granulocytes in the CSF (Supplementary Figure 8B). In contrast, control samples did neither show histopathological signs of inflammation in the ventricles, nor leukocyte extravasation into the CSF (Supplementary Figure 8A).

A detailed flow cytometric analysis of infected brain tissues revealed the presence of a CD45^hi^CD11b^hi^CX3CR1^−^Ly6C^+^ cell population, which might represent either recently immigrated pro-inflammatory monocytes or neutrophils. Neutrophils are characterized by a Ly6C^int^ and Ly6G^+^ phenotype^36^. Their identification by FACS analysis confirmed the histological results and was in line with a significant upregulation of the neutrophil-attracting chemokine *Cxcl2*. Pro-inflammatory monocytes characterized by a Ly6C^hi^ phenotype are known to crucially contribute to the anti-listerial host defense in adult mice^37^. Their mobilization and recruitment mainly depends on the monocyte-attracting cytokines Ccl2 and Ccl7, which also were found at elevated levels in infected brains^16^. In contrast, the number of CD45^lo^CD11b^+^CX3CR1^+^Ly6C^−^ microglial cells increased with age, but no additional infection-induced increase was observed. Microglial cells monitor neuronal activity, support physiological brain development by synaptic pruning and scan their environment for pathological alterations or potential danger signals^38^. Yet, their role during listerial infection in neonates still needs to be elucidated. Notably, neuronal ex vivo *models* have shown that *Lm* readily invades microglial cells^39,40^. We also found that a CD45^hi^CD11b^hi^CX_3_CR1^+^Ly6C^−^ cell subset, which might represent blood-borne anti-inflammatory monocytes (Ly6C^lo^) and/or monocyte-derived M1/2-like macrophages increased after infection. Ly6C^lo^ monocytes are major producers of anti-inflammatory cytokines and promote tissue repair^41,42^. They might thus mediate tissue repair most likely in concert with microglial cells, which can switch their own activation state to an M2-like neuroprotective phenotype^43^. Further work will be required to dissect the inflammatory response in the neonatal brain tissue.

We found that *Lm* invades the olfactory epithelium. A similar cell tropism and route of infection was described for the neurotropic herpes simplex virus (HSV). HSV interacts with its receptors nectin-1 and heparan sulfate, which are mainly expressed at the apical surfaces of olfactory epithelial cells^44^. Also, two facultative intracellular human bacterial pathogens, *Burkholderia pseudomallei* and *Neisseria meningitidis*, penetrate the nasal epithelium and exploit the olfactory nerve bundles to gain access into the CNS. Similar to *Lm*, nasal infection with *B. pseudomallei* and *N. meningitidis* led to focal destruction of the olfactory epithelium. Interestingly, *B. pseudomallei* was able to reach the CNS only after translocation through the olfactory epithelium, although it is also able to infect respiratory epithelial cells^45,46^. Epithelial translocation of *Lm* was not dependent on mucosal barrier disruption. Bacteria were detected within olfactory epithelial cells and associated to nervous axon bundles within the *lamina propria* also in the absence of detectable tissue destruction. A similar phenotype has been described for intestinal translocation of *Lm* in human E-cadherin (hECAD) transgene mice^47,48^.

The *Lm* cytolysin listeriolysin (LLO) encoded by the *hly* gene was shown to play a critical role during the course of infection. LLO deficient bacteria failed to penetrate the olfactory epithelium and to cause local barrier disruption, although individual intraepithelial bacteria could be detected. Consistently, Δ*hly Lm* bacteria were absent from the CNS tissue at later time points. An intermediate phenotype was observed for ActA-deficient *Lm*, as they were still able to penetrate the epithelium and reach the mucosal *lamina propria*, although at significantly lower levels as compared to wild-type bacteria. The reduced translocation might be due to the involvement of ActA in heparan sulfate mediated adhesion and uptake by epithelial cells^49,50^. ActA-independent epithelial translocation might be facilitated by a recently described mechanism exploiting the cellular endocytic machinery to transverse the epithelium^47^. Olfactory sensory neurons exhibit endocytic activities and are able to perform retrograde vesicle transport^51,52^. Indeed, we observed *Lm* within olfactory sensory neurons. *Lm* was also detected in sustentacular cells that might thus also contribute to translocation^51,53^. The known susceptibility of primary neurons to LLO-mediated cell damage might  promote the observed focal epithelial barrier disruption and local recruitment of inflammatory cells^54,55^. Both, migration along or within nervous structures and transport of intracellular *Lm* in migratory macrophages might facilitate translocation into the brain tissue^56,57^. The significantly reduced number of Δ*actA Lm* in CNS tissue favors the hypothesis that *Lm* indeed spreads inside nervous structures since transport in migratory phagocytes to the CNS would not require functional ActA^58^. Also, we detected migration of wild-type *Lm* through the cribriform plate in close proximity of axonal structures. In contrast, Δ*actA* Lm failed to associate with neuronal cell structures and did not reach the brain tissue. This is consistent with findings from Henke et al., who demonstrated *Listeria* actin tail formation within neuronal axons by electron microscopy^59^.

In summary, we present the first murine infection model that reflects human neonatal cerebral listeriosis and allows the analysis of the whole course of the infection. In contrast to previous work that used percutaneous or intracranial bacterial inoculation, our model also allows the analysis of the early steps of the infection and avoids the local tissue damaging effect of intracerebral inoculation^11,60–63^. With the identification of translocation through the olfactory epithelium, spread within the mucosal tissue and ascending infection along neuronal cell structures through the cribriform plate, we identify a previously unrecognized route of *Lm* infection. We also characterize the contribution of important bacterial virulence factors during the various steps of the infection. Future use of this neonatal infection model will allow studies to gain new insights in the microbial pathogenesis and antimicrobial host response of neonatal listeriosis and thus allow the development of novel therapeutic strategies to improve the future clinical management.

## Methods

### Bacterial strains and growth conditions

The bacteria used in this study are *Listeria monocytogenes* (*Lm*) strain EGDe (WT *Lm*), an isogenic LLO deficient strain Δ*hly Lm*, an isogenic InlAB deficient strain Δ*inlA*Δ*inlB Lm* as well as an isogenic ActA-deficient strain *ΔactA Lm*^64–66^. For infection, the *Lm* strains were cultured in brain heart infusion broth (BD) overnight at 30 °C, if required supplemented with 100 µg/mL Spectinomycin (*ΔactA*/pAT28-actA *Lm* and *Δhly*/pAT28-*hly Lm*). Bacteria were washed with PBS, adjusted to OD_600_ 0.95–1 containing ~3–5 × 10^8^ CFU/ml and diluted to the appropriate infection dose. All bacterial mutants were generated by scarless in-frame deletion of genetic regions. To exclude off-target effects, the phenotype of the bacterial mutants (Δ*actA*, Δ*hly*) and their respective complemented strains (Δ*actA+actA*, Δ*hly+hly*) was verified in respect to polar actin polymerization and the presence or absence of a surrounding LAMP1 positive endosomal membrane in cell culture experiments using the murine macrophage cell line J774A.1 (Supplementary Figure 9).

### Cell culture

The murine macrophage cell line J774A.1 (ATCC TIB-67) was routinely cultured in Iscove Basal Medium supplemented with 10% FCS_Hi_ (Biochrom) at 37 °C and 5% CO_2_. For infection, macrophages were seeded in 24-well plates (Corning), containing 12 mm cover glasses (Thermo Scientific). The cell line was authenticated by functional testing, morphology, and growth characteristics. The cell line was free of mycoplasma.

### Infection experiments

Conventionally raised C57BL/6 mice were bred at the Hannover Medical School Animal Facility, the University of Veterinary Medicine Hannover or the Freie Universität Berlin and checked daily for litters. One-day-old male and female mice were used in all experiments. Oral gavage was performed using a 24G silicon catheter (Vygon) with the indicated number of *Lm* in a volume of 10 µl PBS. For intranasal infections, 1 µl of PBS containing the designated amount of *Lm* were applied directly on the nostrils of the neonates. To assess the health status and to avoid excessive stress due to the infection, a scoring system was setup. Grade 1, active: Spontaneous movement, pink skin, the dam cares, pups are placed together and display daily body weight gain. Grade 2, sleepy: Movements to stimulus, pink skin, the dam cares, pups are placed together and daily body weight gain. Grade 3, reduced state: Reduced movements to stimulus, dam cares, pups are placed together and display reduced body weight gain over at least 2 days. Grade 4, severely reduced state: Motionless, dam does not care sufficiently, pups are placed dispersed in the cage and display no body weight gain over at least 2 days. The health status of the infected mice was monitored at least once daily. Termination, if no body weight gain over at least 2 days, insufficiently caring dam (pups placed dispersed) and severely reduced motility to stimulus or starting at grade 3, certainly at grade 4 (Supplementary Figure 10A, B).

At indicated time points, pups were taken randomly via a random number generator out of the cage and bacterial numbers were obtained by collecting blood with a pipette or homogenization of lung, liver, spleen, nasopharynx as well as brain by serial dilution and plating on Blood and Oxford Agar, respectively.

Notably, the same data points for CFU plating of WT *Lm* infections at 1, 3 and 5 dpi in Fig. 1a and Fig. 8a as well as in Figs. 1b and 8i were shown. Data points for the WT *Lm* infections presented in Supplementary Fig 6 A and 6B are also the same data points as in Fig. 1a, b at 3 dpi. Litters analyzed for histopathological manifestations in Fig. 5a, b are identical to the animals analyzed in Supplementary Figure 8A and B, as well as in Fig. 7f, g analyzed for Ly6G^+^ cells in the CNS.

For in vitro co-culture, the respective Lm strain was added to J774A.1 macrophages at a multiplicity of infection (MOI) of 5, followed by centrifugation (250×*g*, 10 min, RT). Thirty minutes after addition of bacteria, cells were washed with PBS and fresh medium containing 50 µg/ml gentamycin was added to prevent extracellular growth of *Lm*. Incubation was carried out for further 60 min. Cells were then rinsed with PBS and cover glasses were transferred to 4% PFA, incubated for 1 h and stored in PBS until further use.

### Immunostaining

Whole heads were fixed with 4% PFA for at least 24 h, dehydrated and embedded in paraffin wax. For immunofluorescence, longitudinally bisected heads were cut into 3 µm thick sections, deparaffinized with xylene and rehydrated in graded ethanol, followed by antigen retrieval for 20 min in 10 mM sodium citrate using a pressure cooker and blocking with 5% BSA/PBS and 10% normal serum for 1 h at RT. Immunostaining was conducted overnight at 4 °C using a primary rabbit anti-Lm antibody (Meridian Life Science, Catalog No.: B65420R, 1:3000), a mouse anti-Tubulin beta III antibody (Merck Millipore, Catalog No.: mab5564, 1:300) to detect neuronal structures, an anti-beta Catenin conjugated with Alexa Fluor647 (Abcam, Catalog No.: ab194119, 1:100) antibody to label cell membranes and cell junctions; combined with appropriate fluorophore conjugated secondary antibodies, donkey anti-rabbit Cy3 (Jackson, Catalog No.: 711-166-152, 1:1000), donkey anti-mouse FITC (Jackson, Catalo No.: 715-096-150, 1:500). Tissues were mounted with Vectashield Mounting Medium with DAPI (Vector Laboratories, Catalog No.: H-1200).

J774A.1 macrophages were permeabilized for 10 min at RT with 0.1% Triton X-100 (Carl Roth). Cells were then blocked with 5% BSA/PBS and 10% normal serum for 1 h at RT. Immunostaining was conducted overnight at 4 °C using a primary rabbit anti-*Lm* antibody, (Meridian Life Science, Catalog No.: B65420R, 1:3000), a primary rat anti-LAMP1 antibody (Abcam, Catalo No.: ab25245, 1:300), MFP-DY-495-Phalloidin (MoBiTec,Catalog No.: MFP-D495-33, 1:50) to label actin; combined with fluorophore conjugated secondary antibodies, donkey anti-rabbit Cy3 (Jackson, Catalog No.: 711-166-152, 1:1000), goat-anti-rat AF647 (Jackson, Catalog No.: 112-605-003, 1:500). Coverslides were mounted with Vectashield Mounting Medium with DAPI (Vector Laboratories, Catalog No.: H-1200).

For analysis of inflammatory distributions in the CNS, collected brains were fixed with 4% PFA for 24 h. Afterwards, fixed brains were incubated overnight with 2% PFA/PBS containing 30% sucrose, embedded in OCT compound (Tissue Tek), cut into 7 μm thick sections (Leica CM3050 S) and stored in a −20 °C freezer. For staining, sections were rehydrated in Tris-buffered saline with 0.05% Tween-20 (Sigma) (named TBST), blocked with 5% rat serum and 10% Fc block (Rat IgG 2b kappa; clone: 2.4G2) diluted in 1× TBST. *Lm* was labeled using a rabbit anti-*Lm* (Meridian Life Science, Catalog No.: B65420R, 1:3000), a mouse anti-CD45 antibody conjugated with PE (BioLegend, Catalog No.: 103106, 1:100) or conjugated with APC (BD Bioscience, Catalog No.: 559864, 1:100) to label most leukocytes, an rat anti-CD11b antibody conjugated with PE to label myeloid cells (BD Bioscience, Catalog No.: 553311, 1:100). The FITC-conjugated goat-anti-rabbit antibody (Jackson, Catalog No.: 111-096-045, 1:100) was applied as secondary antibody.

### Confocal and Immunofluorescence microscopy

Confocal microscopy of bisected head sections was performed with a LSM 880 confocal laser-scanning microscope with a Plan-Apochromat 40x/1.3 Oil DIC M27 and a Plan-Apochromat 63x/1.4 NA objective, driven by Zen 2.1 software (Carl Zeiss, Jena, Germany). Image stacks were deconvolved using Huygens® Essential 15.10 (Scientific Volume Imaging, Hilversum, NL) and maximum intensity projections (MIPs) were calculated for display purposes and adjusted for brightness and contrast in ImageJ/Fiji (NIH, USA) and ZEN Lite 2.3 (Carl-Zeiss, Jena, Germany). Immunofluorescence microscopy of bisected head sections was performed with a Axio Imager M2 with a Plan-Apochromat 10x/0.3, a Plan-Apochromat 20x/0.8 M27 and a Plan-Apochromat 40x/0.95 Korr M27 objective (Carl Zeiss, Jena, Germany), driven by Zen 2.1 software (Carl Zeiss, Jena, Germany). Immunofluorescence microscopy of entire brains was performed on a Zeiss Axiovert 200 M Inverted Microscope with a Plan-NEOFLUAR 5x/0.16 M27 and a Plan-Apochromat 20x/0.8 M27 objective (Carl-Zeiss, Jena, Germany), driven by AxioVision 4.8. Immunofluorescence microscopy of J774A.1 macrophages was conducted on a Leica DMI 6000B Inverted Microscope with a 63x HCX PL APO oil objective (Leica Microsystems, Wetzlar, Germany), driven by LAS X 3.3.0.16799 software (Leica Microsystems, Wetzlar, Germany). Image stacks were deconvolved using the LAS X 3.3.0.16799 software (Blind deconvolution, Auto Quant^Tm^ Deconvolution algorithm). Images were processed using AxioVision 4.8, ZEN Lite 2.3 software (Carl-Zeiss, Jena, Germany), LAS X 3.3.0.16799 software (Leica Microsystems, Wetzlar, Germany) and ImageJ Fiji (NIH, USA). Pictures were adjusted for brightness and contrast.

### Transmission electron microscopy

Samples were fixed with 4% PFA in PBS and send in the fixative. After several washing steps with PBS samples were further fixed with 1% osmiumtetroxide in PBS for 1 h at room temperature (RT). After a washing step with PBS samples were dehydrated with 10%, 30%, and 50% acetone on ice before incubation in 70% acetone with 2% uranylacetate for overnight at 7 °C. Samples were further dehydrated with 90 and 100% acetone on ice, allowed to reach room temperature and further dehydrated with 100% acetone, then changed into 100% ethanol. Subsequently, samples were infiltrated with the epoxy resin Low Viscosity resin (Agar Scientific, Stansted, UK). After polymerization for 2 days at 75 °C ultrathin sections were cut with a diamond knife, collected onto butvar-coated 3000 mesh grids, and counterstained with 4% aqueous uranylacetate for 4 min. Samples were imaged in a Zeiss TEM 910 at an acceleration voltage of 80 kV and at calibrated magnifications. Images were recorded digitally at calibrated magnifications with a Slow-Scan CCD-Camera (ProScan, 1024 × 1024, Scheuring, Germany) with ITEM-Software (Olympus Soft Imaging Solutions, Münster, Germany). Contrast and brightness were adjusted with Adobe Photoshop CS5 and afterwards sharpened with Image J Fiji (NIH, USA).

### Histology and pathological assessment

Entire heads were longitudinally sectioned, fixed in 4% PFA for at least 24 h. Subsequently, tissue was decalcified for 48 h in 10% disodium-ethylenediaminetetraacetate (EDTA), embedded in paraffin, sectioned at a thickness of 5 µm and stained with hematoxylin and eosin for histological examination.

Immunohistochemistry was performed using a monoclonal rat anti-mouse Ly6G-specific antibody (Catalog No.: 127602; BioLegend; 1:500). Paraffin sections were rehydrated in graded ethanol. For blocking of the endogenous peroxidase, formalin-fixed, paraffin-embedded tissue sections were treated with 0.5% H_2_O_2_ diluted in methanol for 30 min at room temperature (RT). Subsequently, sections were heated in 10 mM sodium citrate buffer pH 6.0 for 20 min in a microwave oven (800 W). Following incubation with 20% goat serum each for 30 min to block non-specific binding sites, sections were incubated with the primary antibody for 1.5 h at RT. Thereafter, sections were treated for 30 min at RT with the secondary antibody biotinylated rabbit anti-rat immunoglobulin (Catalog No.: BA 4001; Vector Laboratories; 1:200). Slides were subsequently incubated with the peroxidase-conjugated avidin-biotin complex (Catalog No.: PK 6100; Vector Laboratories) for 30 min at RT. All antibodies were diluted in PBS. After visualization of the positive antigen-antibody reaction by incubation with 3.3-diaminobenzidine-tetrachloride (DAB) for 5 minutes, sections were counterstained with hematoxylin.

To compare the histopathological pictures of infected mice, a scoring system was setup. Histopathological pictures were classified as “negative” with a score of 0, “mild” symptoms received a score of 2, “moderate” symptoms a score of 4, and “severe” symptoms a score of 6. An additional assessment of “multifocal” or “necrotizing” increased the respective score by 1 point. The histopathological evaluation was performed in a blinded fashion.

### Gene expression analysis

Total RNA was isolated from FastPrep homogenized brains using TRIzol (Ambion) and RNA concentration was determined with a Spectramax i3x device (Molecular Devices). Complementary DNA (cDNA) was synthesized from 5 µg RNA with Oligo(dT)18 primers and ReverdAid reverse transcription (Fermentas) for quantitative RT–PCR. Gene expression was then determined using QPCR Rox mix (Thermo Scientific) with cDNA and TaqMan probes Hprt (Thermo Fisher, Catalog No.: Mm00446961_m1, 1:20), Tnfa (Thermo Fisher, Catalog No.: Mm00443258_m1 1:20), Cxcl2 (Thermo Fisher, Catalog No.: Mm00436450_m1, 1:20), Ccl2 (Thermo Fisher, Catalog No.: Mm00441242_m1, 1:20), Ccl7 (Thermo Fisher, Catalog No.: Mm00443113_m1, 1:20) and samples were run on a Mx3005P qPCR system (Stratagene, Agilent Technologies). Transcript results were normalized to the housekeeping gene Hprt and expressed as fold change.

### Isolation of immune cells from the CNS

Neonatal mice were anesthetized with 0.12 mg ketamin (100 mg/ml) and 0.004 mg xylazine (20 mg/ml) per gram body weight and transcardially perfused with PBS. Whole brains were then collected and digested with the Neural Tissue Dissociation Kit P (Miltenyi Biotec, Catalog No.: 130-092-628) and a gentleMACS Dissociator (Milteny Biotec) according to manufacturer’s instructions. The brain homogenates were then separated using a 70–37–30% percoll gradient and cells between the 70 and 37% layer were collected.

### Flow cytometry

Single-cell suspensions for fluorescence-activated cell sorting (FACS) were stained with a mouse anti-CD45.2 antibody conjugated with Pacific Blue (BioLegend, Catalog No.: 109820, 1:1000) to label leukocytes, an rat anti-CD11b antibody conjugated with APC-Cy7 (BD Pharmingen, Catalog No.: 557657, 1:1000) to label myeloid cells, a mouse anti-CX3CR1 antibody conjugated with BV510 (BioLegend, Catalog No.: 149025, 1:500) to label microglia/Ly6C^-^-monocytes/alternatively activated macrophages, a rat anti Ly6C antibody conjugated with AF700 (BioLegend, Catalog No.: 128023, 1:1000) to label inflammatory monocytes as well as a rat anti-mouse Ly6G antibody conjugated with PE/Cy7 to label neutrophils (BioLegend, Catalog No.: 127617, 1:500). Cells were stained for 20 min at 4 °C. The data were acquired on a LSR II flow cytometer driven by FACSDIVA 6.1.1 software (BD Bioschiences) and analyzed with FlowJo software 10.4 (Tree Star). The gating strategy utilized during this study is depicted in Supplementary Figure 11.

### Ethics statement

All animal experiments were performed in compliance with the German animal protection law (TierSchG) and approved by the local animal welfare committee (approval 12/0693 and 14/1385 of the Niedersächsisches Landesamt für Verbraucherschutz und Lebensmittelsicherheit Oldenburg, Germany, and G0304/15 of the Landesamt für Gesundheit und Soziales Berlin, Germany).

### Statistical analysis

Sample sizes were chosen to reach statistical significance (*p* < 0.05) for a pre-specified effect based on variations observed in former studies. No statistical method was used for sample size estimation in animal experiments. Upon infection, pups were taken randomly from the litter by applying a random number generator; no blinded analyses were employed. Indicated mouse numbers display biological replicates and are in most cases derived from different litters to reflect inter-litter variations. The Graphpad Prism 7 software (Version 7.01) was used for the statistical analysis. Medians are shown if not indicated otherwise. The unpaired, two-tailed Mann–Whitney test was used for comparison of two groups and the one-way ANOVA Kruskal-Wallis with Dunnets’ post test was used for comparison of three or more groups. *P*-values are indicated as follows: *p < 0.05, **p < 0.01, ***p < 0.001, ****p < 0.0001; n.s.; not significant, *p* > 0.05. All bacterial plating and RT–PCRs were performed in duplicates. Histopathological examination was performed blinded for age and infection status.

## Electronic supplementary material

Supplementary Information

## Data Availability

Additional data that support the findings of this study are available from the corresponding authors upon request.
