# Supplementary material for: The olfactory epithelium as a port of entry in neonatal neurolisteriosis
Source: Nat Commun. 2018 Oct 15;9:4269. doi: 10.1038/s41467-018-06668-2 (PMC6189187; doi:10.1038/s41467-018-06668-2)
Supplement: Supplementary file 1 — Supplementary Information [file 41467_2018_6668_MOESM1_ESM.pdf]

Supplementary Information

The olfactory epithelium as a port of entry in neonatal neurolisteriosis

Pägelow et al.

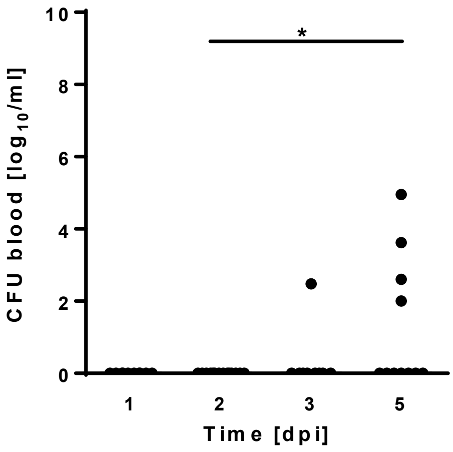

Supplementary Figure 1 | Rare bacteremia during i.n. induced neurolisteriosis. Colony counts in blood samples after i.n. infection. One-day-old mice were infected with 1 x 10^4 CFU *Lm* and blood samples were taken at 1, 2, 3 and 5 dpi. Each dot defines an individual mouse with n= 10 pups (1 dpi), n= 14 (2 dpi), n=9 (3 dpi), n= 11 (5 dpi); median; one-way ANOVA Kruskal-Wallis with Dunnets‘ post test; * $p < 0.05$

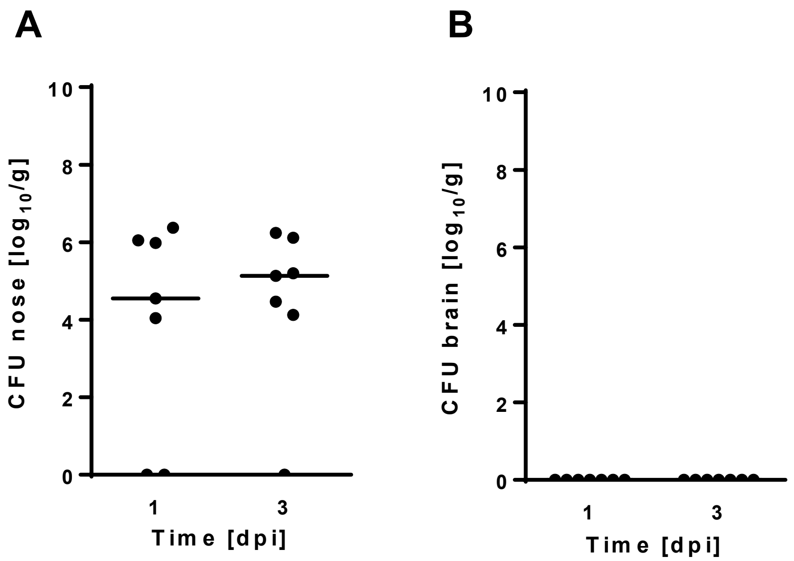

Supplementary Figure 2 | CNS invasion via the i.n. route is age-dependent. Bacterial counts in nasopharyngeal mucosal tissue (**A**) and total brain tissue (**B**) at 3 dpi. Eleven-day-old mice were infected i.n. with 2 x 10^5 CFU *Lm*. Each dot defines an individual mouse with n= 7 pups at 1 and 3 dpi; median.

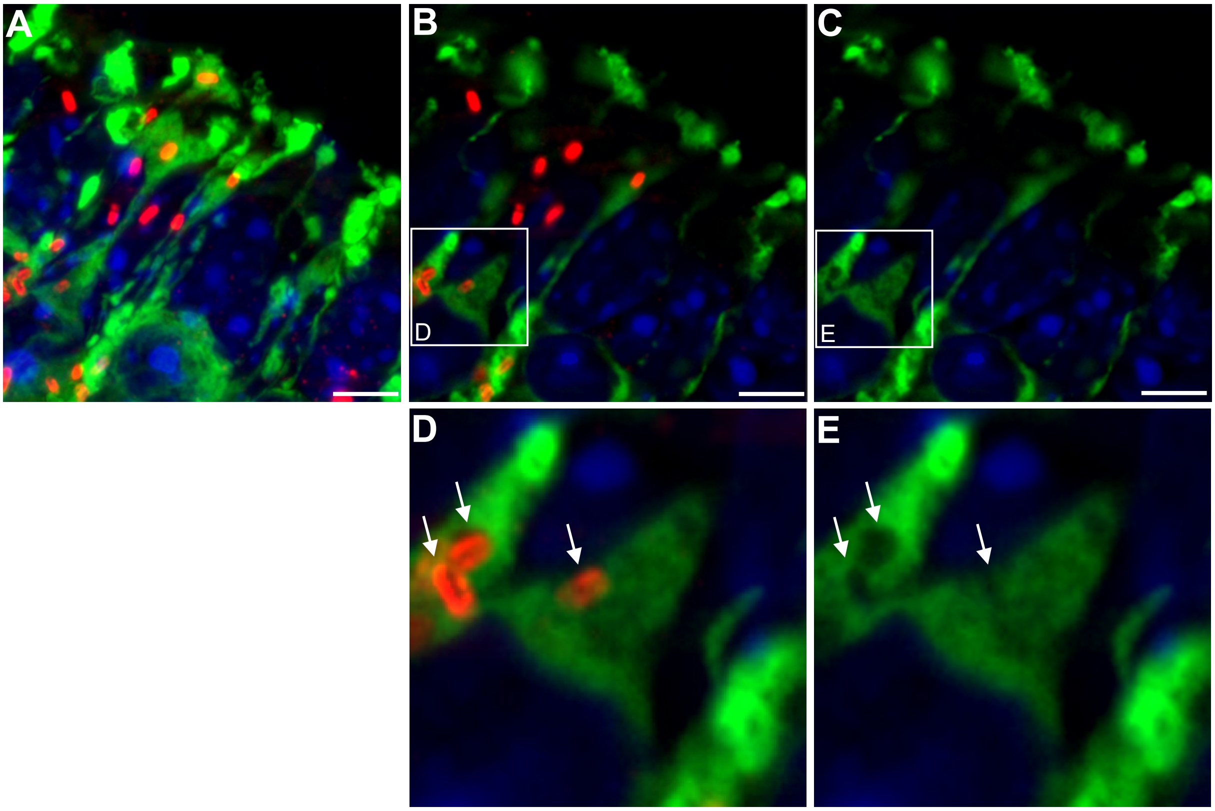

Supplementary Figure 3 | *Lm* co-localizes with olfactory sensory neurons. Immunostaining of olfactory sensory neurons and Lm after i.n. infection of 1-day-old with 1 x 10^7 CFU *Lm*. *Lm* (red), neuronal marker β-tubulin III (green) and DNA (DAPI, blue). (**A**) Surpass view of a Z-stack image of the olfactory epithelium at 1 dpi showing *Lm* in the olfactory epithelium and associated with neuronal cellular structures. (**B** and **C**) Slice view plane Z1 of the initially acquired Z-stack image, with and without the red channel. (**D** and **E**) Zoom views of (B) and (C); white arrows indicate bacteria associated with neuronal cellular structures (scale bar 5 µm). The images are representative of n= 6 pups.

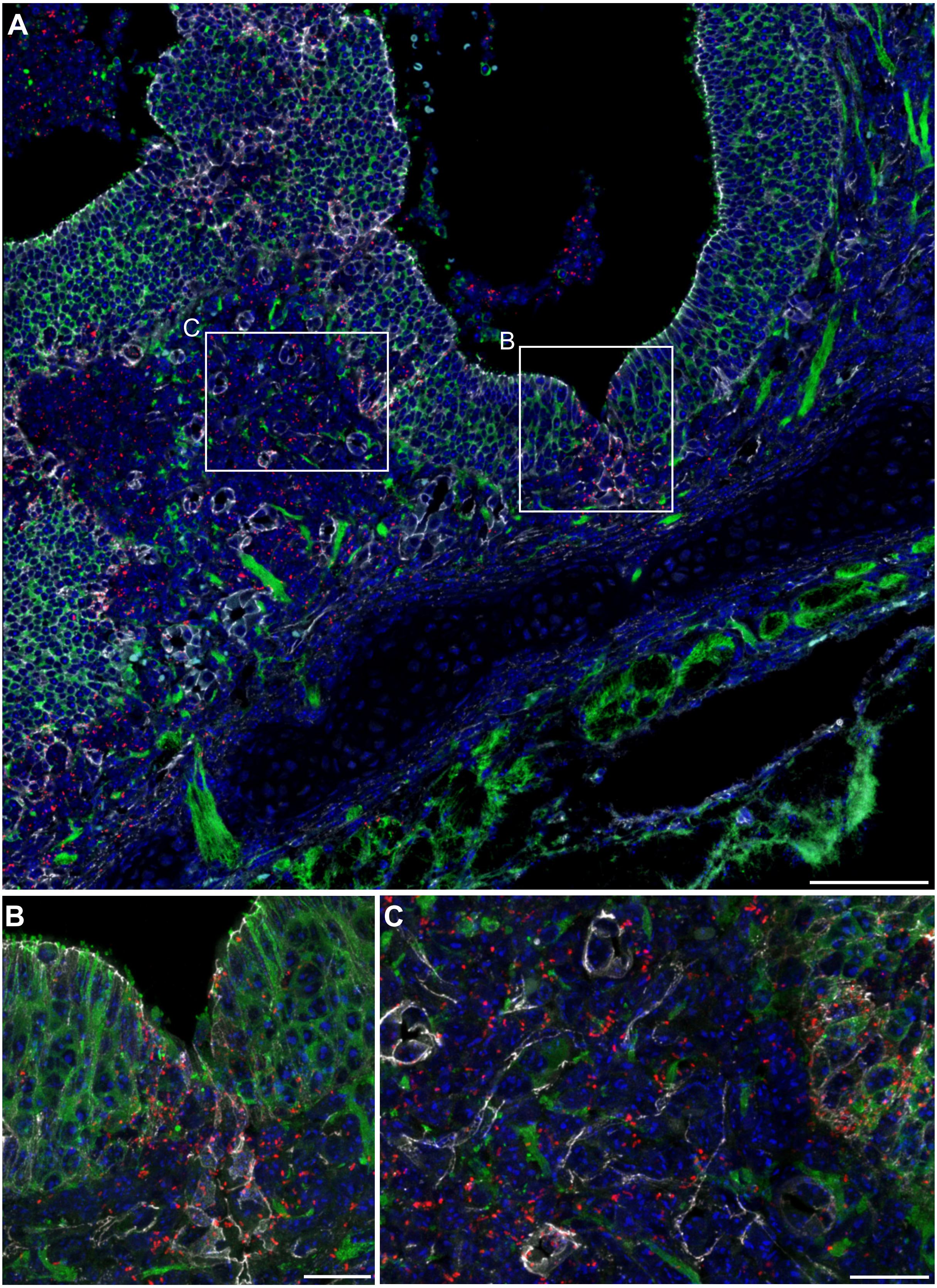

Supplementary Figure 4 | Intranasal infection causes destruction of the olfactory mucosa. Immunostaining of the olfactory system after i.n. infection of 1-day-old mice with 1 x 10^7 CFU *Lm*. Sections were stained for *Lm* (red), neuronal marker β-tubulin III (green), the tight-junction marker β-catenin (white) and DNA (DAPI, blue). (**A**) Sagittal view at 1 dpi showing focal disruption of the olfactory epithelial integrity (scale bar 100 µm). (**B** and **C**) Enlarged views of framed inserts in (A) (scale bar 20 µm). The images are representative of n= 8 pups.

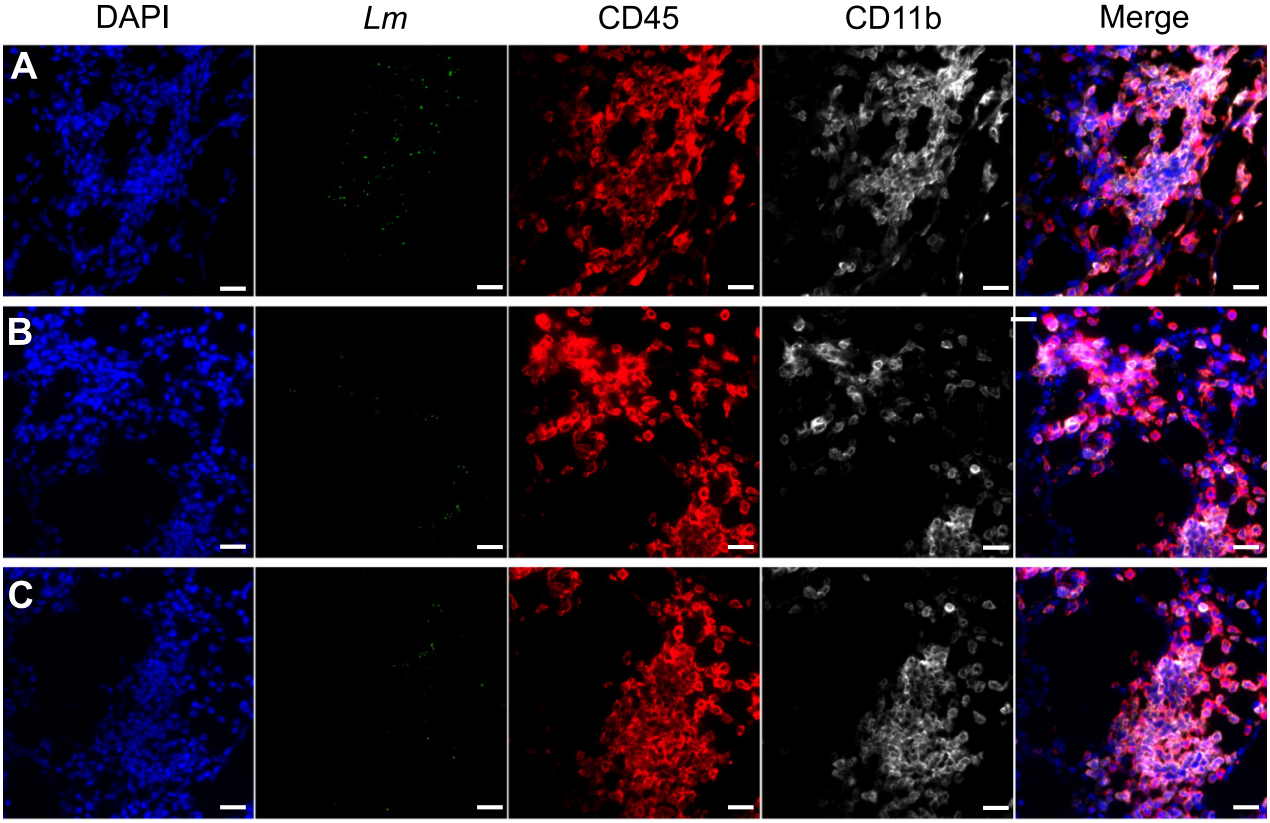

Supplementary Figure 5 | Recruitment of immune cells to sites of cerebral infection. One-day-old mice were inoculated i.n. with 1 x 10^5 CFU *Lm*. (**A**-**C**) Mice were sacrificed at 3 dpi and olfactory bulbs were processed for immunostaining. *Lm* (red), leukocytes (CD45, red), myeloid cells (CD11b, white) and DNA (DAPI, blue) (scale bar 20 µm). The pictures are representative of n= 6 pups**.**

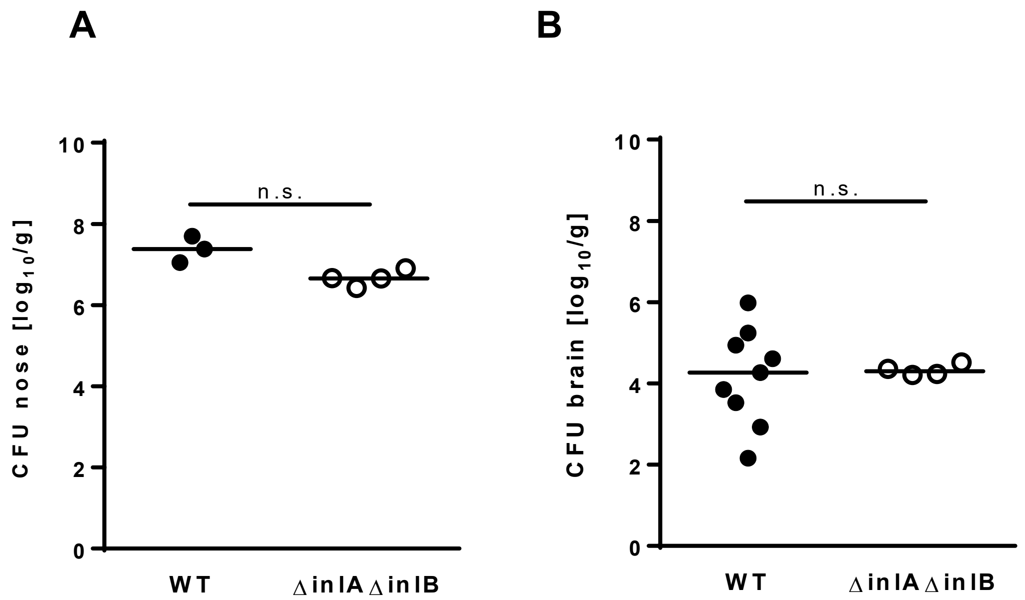

Supplementary Figure 6 | InlA/B are expendable for CNS invasion via the i.n. route. Bacterial counts in nose (**A**) and total brain tissue (**B**) after i.n. infection of 1-day-old mice with 1 x 10^4 CFU WT or *ΔinlAΔinlB Lm* at 3 dpi. Each dot defines an individual mouse. Nose: WT *Lm* n= 3, *ΔinlAΔinlB Lm* n= 4; brain WT *Lm* n= 9, *ΔinlAΔinlB* Lm n= 4. Median; unpaired, two-tailed Man-Whitney test; n.s. $p > 0.05$.

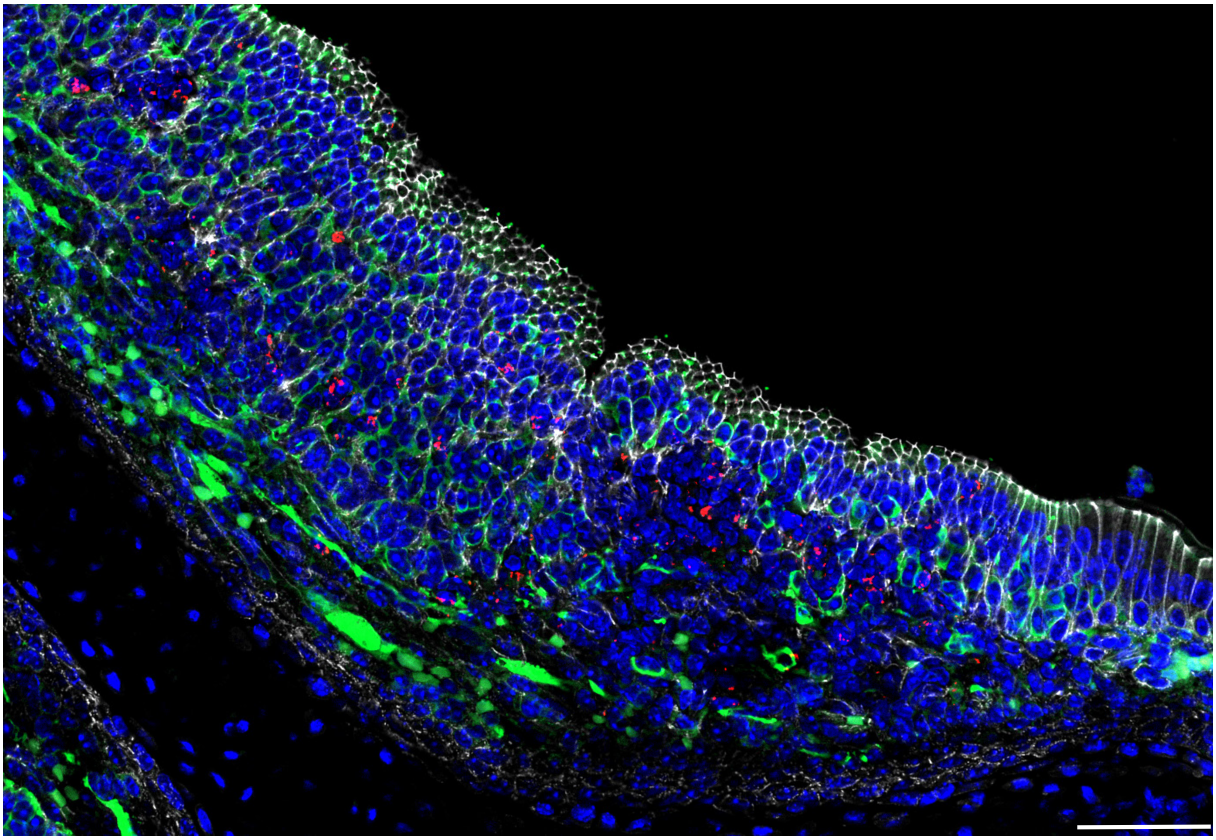

Supplementary Figure 7 | Dissemination of ∆*actA Lm* within the olfactory mucosa. Immunostaining of the olfactory mucosa at 1 dpi after i.n. infection of 1-day-old mice with 1 x 10^7 CFU ∆*actA Lm*. *Lm* (red), the neuronal marker β-tubulin III (green), the tight junction marker β-catenin (white) and DNA (DAPI, blue). Shown is ∆*actA Lm* in the olfactory epithelium and *lamina propria* at 1 dpi (scale bar 50 µm). The images are representative for samples from n= 6 pups.

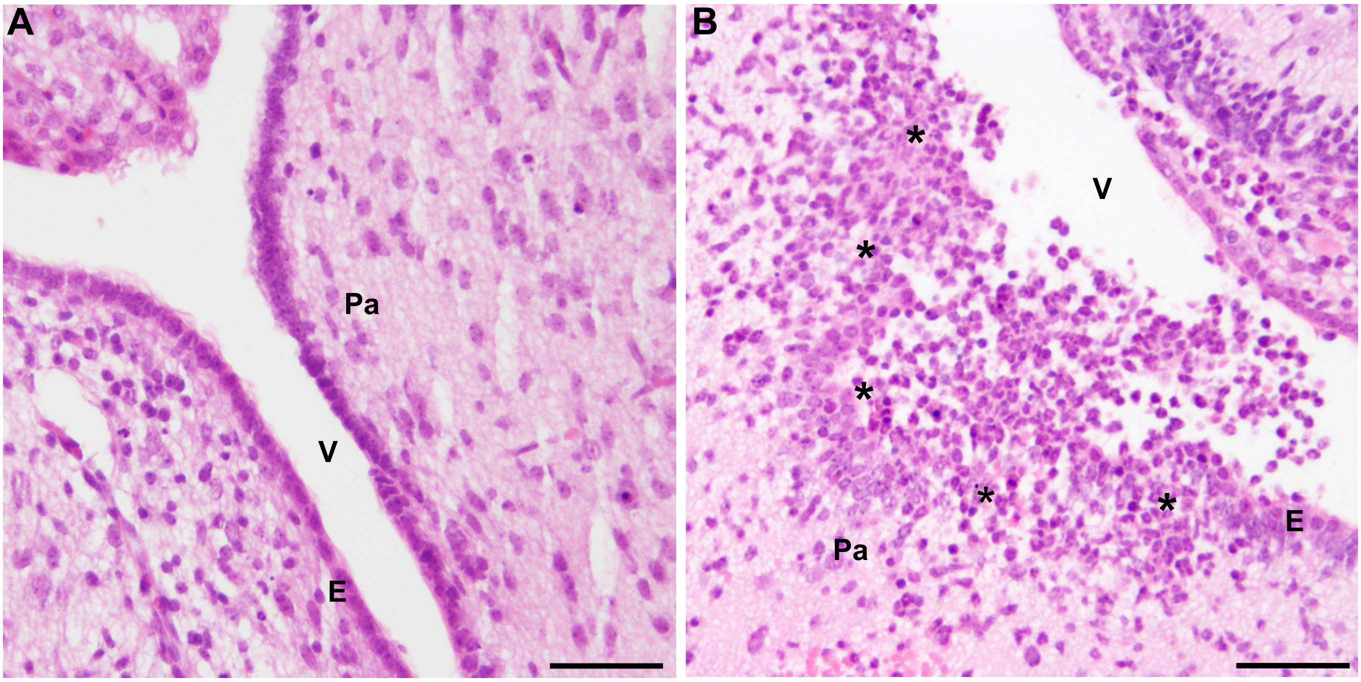

Supplementary Figure 8 | CNS infection induces a neutrophilic ventriculitis. One-day-old mice were infected i.n. with 1 x 10^4 CFU *Lm* or left untreated. Tissue was taken at 5 dpi or from age-matched control animals. HE-staining of periventricular parenchyma (Pa), ependyma (E), ventricle (V). (**A**) Sagittal view of the ventricular system of a 6-day-old control mouse. (**B**) Sagittal view of the same brain region, asterisks mark inflammatory infiltrates (scale bar 50 µm). Images are representative for samples from n= 6 pups; images of age-matched controls are derived from n= 4 pups.

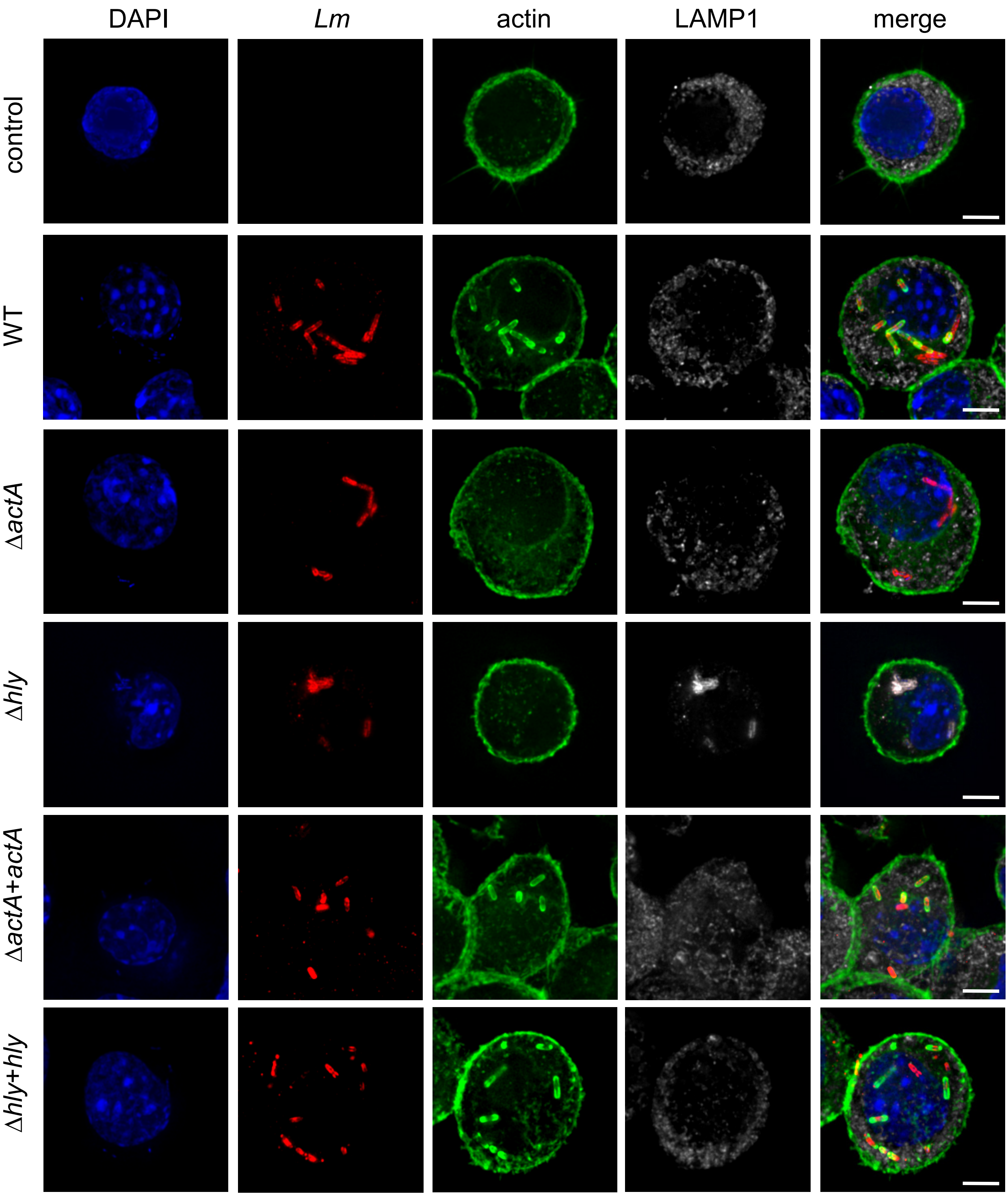

Supplementary Figure 9 | In-frame deletions of ActA and LLO do not induce polar effects. Immunostaining of J774A.1 control macrophages or after infection with wt, *actA*- and *hly*-deficient *Lm* as well as respective complemented strains at 90 min post infection. Cells were stained for *Lm* (red), actin (green), LAMP1 (gray) and DNA (DPAI,blue) (scale bar 5 µm). The pictures are representative of two independent experiments.

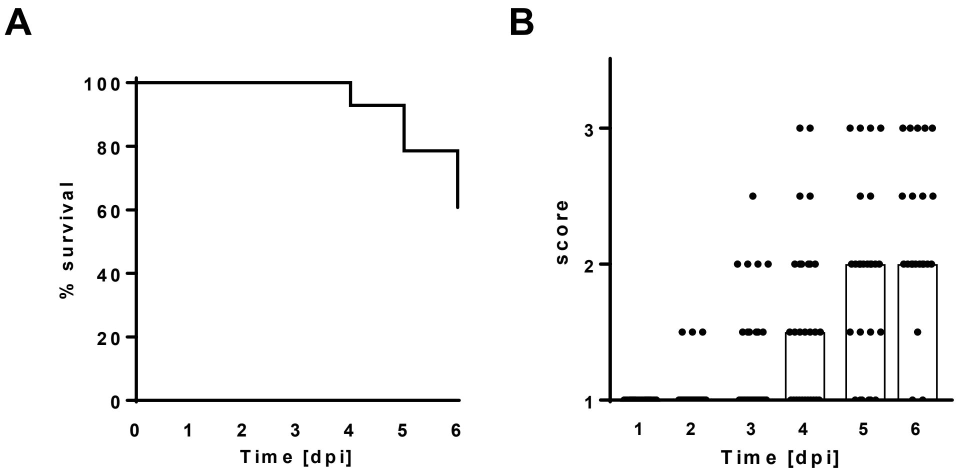

Supplementary Figure 10 | Survival and morbidity. One-day-old mice were given 1 x 10^4 WT *Lm* i.n. and sacrificed when reaching a defined clinical score as described in the method section. (**A**) Survival rates of neonatal mice after i.n. infection with *Lm*. (**B**) Respective score of the infected mice in (A). Each dot defines an individual mouse (n= 28); median.

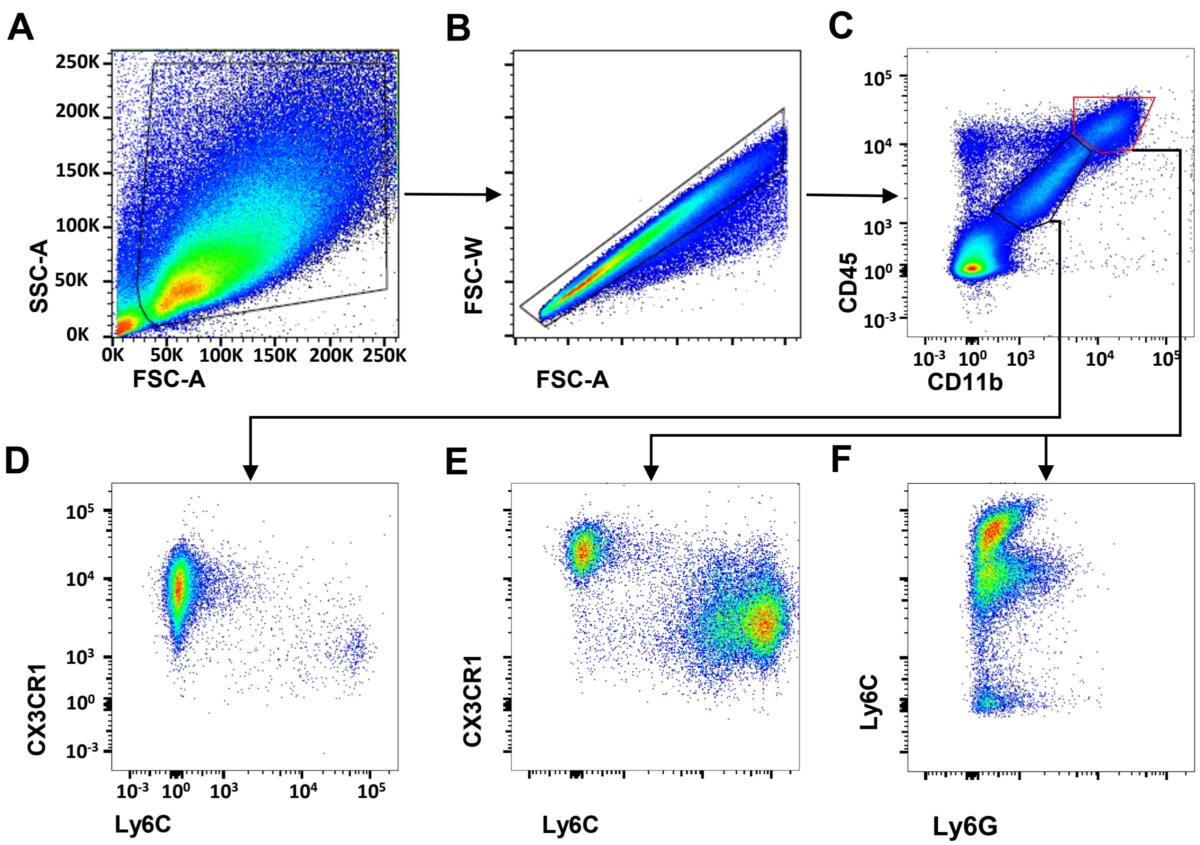

Supplementary Figure 11 | Gating strategies used for flow cytometry. Gating strategies used to analyze immune cells in the CNS of neonatal mice upon i.n. infection with WT *Lm* as described in the methods section and shown in Fig. 7. (**A**) To separate cell debris from intact cells, suspensions were first gated according to their cell-surface area or size (Forward-scatter, FSC-A) and their granularity or internal complexity (Side-scatter, SSC-A). (**B**) Cell multiplets were excluded from the initially gated population by repeatedly using the FSC-A and the FSC-W (width) parameter. (**C**) The single cell population was gated on the common leukocyte marker CD45 as well as for the pan-myeloid marker CD11b. $CD45^{lo}CD11b^{+}$ cells (black polygon in **C**), as well as $CD45^{hi}CD11b^{hi}$ (red polygon in **C**) were gated on CX3CR1 and Ly6C as shown in Fig. 7D. $CD45^{hi}CD11b^{hi}$ cells were additionally gated on Ly6C and Ly6G as shown in Fig. 7E.

Supplementary Table 1 | Histopathological manifestation in the nasopharynx.

		WT *Lm*		Δ*actA Lm*	
Time	Mouse	Symptoms	Score	Symptoms	Score
1 dpi	1	none	0	b	3
	2	none	0	none	0
	3	b	5	a	2
	4	b	5	-	-
	5	c, d	5	-	-
	6	b	5	-	-
3 dpi	1	c, d, e, h	6	b	3
	2	c, d, e, f	6	b	5
	3	c, d, e	6	b	5
	4	c,g	6	-	-
	5	none	0	-	-
	6	c, d, e	6	-	-
	7	c, d, e	8	-	-
	8	c, d, e	6	-	-
5 dpi	1	c, d, e, f, i	6	b	5
	2	c, d, e	8	b	5
	3	c, d, e, f, i	6	b	5
	4	c, d, e, f	8	-	-

One-day-old mice were infected i.n. with 1 x 10^4 CFU WT *Lm* or Δ*actA Lm*. Tissue was harvested at 1, 3 and 5 dpi. Quantitative histopathological score as defined in the methods section. a: focal, purulent rhinitis; b: multifocal, purulent rhinitis; c: multifocal, purulent-necrotizing rhinitis; d: purulent-necrotizing inflammation of adjacent connective tissue; e: purulent-necrotizing inflammation of adjacent cartilage; f: purulent-necrotizing inflammation of adjacent bones; g: focal, purulent lymphadenitis; h: focal, purulent *otitis interna*; i: purulent-necrotizing glossitis.

Supplementary Table 2 | Histopathological manifestation in the CNS.

		WT *Lm*		Δ*actA Lm*	
Time	Mouse	Symptoms	Score	Symptoms	Score
1 dpi	1	none	0	none	0
	2	none	0	none	0
	3	none	0	none	0
	4	none	0	-	-
	5	none	0	-	-
	6	none	0	-	-
3 dpi	1	none	0	none	0
	2	a	2	none	0
	3	c	4	none	0
	4	c	4	-	-
	5	none	0	-	-
	6	a	3	-	-
	7	b	3	-	-
	8	c	3	-	-
5 dpi	1	c	5	none	0
	2	c	5	none	0
	3	c	5	none	0
	4	c	5	-	-

One-day-old mice were infected i.n. with 1 x 10^4 CFU WT *Lm* or Δ*actA Lm*. Tissue was harvested at 1, 3 and 5 dpi. Quantitative histopathological score as defined in the methods section. a: focal, purulent meningitis; b: focal, purulent meningoencephalitis; c: focal, purulent-necrotizing meningoencephalitis.
